# The Influences of Macronutrients on Bone Mineral Density, Bone Turnover Markers, and Fracture Risk in Elderly People: A Review of Human Studies

**DOI:** 10.3390/nu15204386

**Published:** 2023-10-16

**Authors:** Minkyung Je, Kyeonghoon Kang, Jun-Il Yoo, Yoona Kim

**Affiliations:** 1 Department of Food and Nutrition, Gyeongsang National University, 501 Jinju-daero, Jinju 52828, Republic of Korea; alsrud4687@naver.com (M.J.); pos26109@gmail.com (K.K.); 2 Department of Orthopaedic Surgery, Inha University Hospital, 27 Inhang-Ro, Incheon 22332, Republic of Korea; furim@hanmail.net; 3 Department of Food and Nutrition, Institute of Agriculture and Life Science, Gyeongsang National University, 501 Jinju-daero, Jinju 52828, Republic of Korea

**Keywords:** macronutrients, bone mineral density, bone turnover, fracture and elderly people

## Abstract

Osteoporosis is a health condition that involves weak bone mass and a deteriorated microstructure, which consequently lead to an increased risk of bone fractures with age. In elderly people, a fracture attributable to osteoporosis elevates mortality. The objective of this review was to examine the effects of macronutrients on bone mineral density (BMD), bone turnover markers (BTMs), and bone fracture in elderly people based on human studies. A systematic search was conducted in the PubMed^®^/MEDLINE^®^ database. We included human studies published up to April 2023 that investigated the association between macronutrient intake and bone health outcomes. A total of 11 meta-analyses and 127 individual human studies were included after screening the records. Carbohydrate consumption seemed to have neutral effects on bone fracture in limited studies, but human studies on carbohydrates’ effects on BMD or/and BTMs are needed. The human studies analyzed herein did not clearly show whether the intake of animal, vegetable, soy, or milk basic proteins has beneficial effects on bone health due to inconsistent results. Moreover, several individual human studies indicated an association between eicosapentaenoic acid (EPA), docosahexaenoic acid (DHA), and osteocalcin. Further studies are required to draw a clear association between macronutrients and bone health in elderly people.

## 1. Introduction

Osteoporosis is a skeletal disorder characterized by decreased bone mass and microarchitecture, leading to an increased risk of fragility fractures of the hip, spine, and other skeletal sites, which is an emerging global public health problem as the population ages [1,2,3]. In 2010, 5.5 million men and 22 million women in Europe [4,5], as well as 10.2 million United States (US) residents [6] aged over 50, were affected by osteoporosis. Interacting risk factors, such as clinical (low peak bone mass and hormonal factors), medical (the use of certain drugs, e.g., glucocorticoids), behavioral (smoking and low physical activity), nutritional, and genetic (race, small body size, and a personal or family history of fracture) variables are attributable to an elevated risk of osteoporotic fracture [7,8]. According to the World Health Organization (WHO) [1], osteoporosis is defined as a bone mineral density (BMD) of 2.5 or more standard deviations (SDs) below peak bone mass, and osteopenia is defined as bone mass between 1.0 and 2.5 SDs below peak.

A modification of lifestyle factors (e.g., nutrition, exercise, smoking, alcohol intake, and sun exposure) to maximize peak bone mass and strength is a crucial approach for the prevention of osteoporosis or low bone mass later in life [8,9,10,11,12]. In particular, nutritional aspects are one of the modifiable factors in the accumulation and maintenance of bone mass as well as bone loss prevention and treatment [13].

### 1.1. Current Position on Calcium and Vitamin D Supplementation for Fracture Risk

Bone strength reflects the integration of two main features: bone density and bone quality. A meta-analysis by Reid et al. (2014) [14] showed no significant effect of vitamin D on BMD in either the spine or the total hip, but there were small favorable effects on BMD at the femoral neck (FN) (weighted mean difference (WMD) 0.8%; 95% confidence interval (CI) 0.2 to 1.4) with heterogeneity among trials (*I*^2^ = 67%, P_het_ < 0.00027). According to recommendation statements of the US Preventive Services Task Force, vitamin D supplementation alone or with calcium does not reduce the risk of fracture in healthy community-dwelling adults [15]. In line with this, the International Osteoporosis Foundation supported the notion that calcium supplementation with vitamin D could prevent future fracture risk in individuals at high risk of calcium and vitamin D insufficiency as well as in those undergoing osteoporosis treatment. Moreover, meta-analyses indicated that vitamin D supplementation without calcium is not associated with a reduced risk of fracture [16,17,18], while that with calcium is associated with fracture prevention [16,17,18,19].

A recent meta-analysis of 11 randomized controlled trials (RCTs) [20,21,22,23,24,25,26,27,28,29,30] of 34,243 subjects conducted by Yao et al. (2019) [16] showed that vitamin D supplementation alone (daily or an intermittent dose of 400–30,000 IU) was not associated with a decreased risk of any fracture or hip fracture. However, combined supplementation with vitamin D at 400–800 IU per day and calcium at 1000–1200 mg per day was associated with a decreased risk of any fracture (rate ratio = 0.94; 95% CI 0.89 to 0.99) and hip fracture (rate ratio = 0.84; 95% CI 0.72 to 0.97) in a meta-analysis of six RCTs [28,31,32,33,34,35] (49,282 subjects). In a meta-analysis of 11 RCTs [28,31,32,33,34,35,36,37,38,39,40] conducted by Chung et al. (2011) [19], combined vitamin D and calcium supplementation reduced the fracture risk (pooled relative risk (RR) = 0.88; 95% CI 0.78 to 0.99) in older adults. However, the finding changed based on the study settings (RR = 0.71; 95% CI 0.57 to 0.89) compared with a community-dwelling setting (RR = 0.89; 95% CI 0.76 to 1.04).

In a meta-analysis of 33 RCTs [20,24,25,26,27,28,30,32,33,35,36,39,41,42,43,44,45,46,47,48,49,50,51,52,53,54,55,56,57,58,59,60,61] with 51,145 older adults conducted by Zhao et al. (2017) [62], no association between calcium (risk ratio = 1.53; 95% CI 0.97 to 2.42), vitamin D (risk ratio = 1.21; 95% CI 0.99 to 1.47), or combined calcium and vitamin D (risk ratio = 1.09; 95% CI 0.85 to 1.39) supplements and hip fracture was observed compared with placebo or no treatment.

### 1.2. The Association between Bone Mineral Density and Bone Turnover Markers

An increasing number of studies are showing inverse associations between BMD values and bone turnover markers (BTMs; resorption and formation) [63,64]. Only BMD measurements are insufficient to predict fracture risk. BTMs can be complementary parameters even though they are independent parameters to evaluate fracture risk [63]. The inverse association between BMD and BTMs is positively associated with aging and early menopause [64].

Bone turnover markers (BTMs) are biomarkers that can be measured in the blood and/or urine [65]. They can be used to effectively assess bone status in the short term. Bone is a metabolic structure that is continuously remodeled through bone resorption after peak bone mass is reached during life [66,67]. BTMs can be classified into markers of bone formation (e.g., osteocalcin (OC), bone alkaline phosphatase (BALP), and type 1 procollagen-N-propeptide (P1NP)) and bone resorption (e.g., C-terminal telopeptide cross-link of type 1 collagen (CTX), N-terminal of type 1 collagen (NTX), and deoxypyridinoline (DPD)) [68,69]. In particular, P1NP and CTX are commonly measured as BTMs [63,66].

### 1.3. The Association between Macronutrients and Bone Metabolism

Among the numerous functions of macronutrients in our body, one of the metabolisms of carbohydrate and fat related to bone is peroxisome proliferator-activated receptor γ (PPARγ). PPARγ is instrumental in regulating fat and glucose metabolism, and its activation also exerts profound effects on bone metabolism.

The possibility of a positive interaction between dietary protein and bone health is uncertain. Dietary protein uptake can promote enteric calcium absorption, insulin-like growth factor-1 (IGF-1), and the growth of muscle mass and strength as well as restrain parathyroid hormone (PTH) [70,71,72,73,74,75]. Several studies have reported that high dietary protein or dietary acid load can contribute to increased urinary calcium excretion and a reduction in calcium reabsorption [73,76,77,78,79,80]. Consistent with this, differences in PTH and calcitriol were not observed in RCTs [81,82,83,84] despite protein quantity.

### 1.4. The Objective of This Review

Given the current evidence on calcium and vitamin D supplementation for fracture risk, PPARγ involved in glucose and fat metabolism, and IGF-1 involved in protein metabolism; this review aimed to clarify the effects of carbohydrate, fat, and protein on bone-health-related markers in elderly people with a focus on human studies.

## 2. Methods

**Figure 1:**
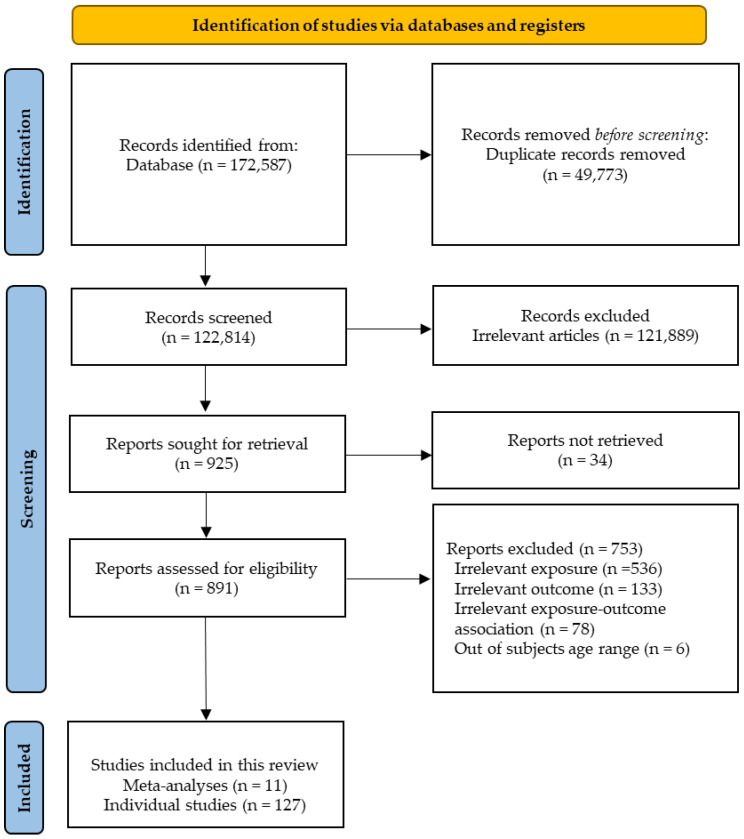
The flow diagram of selection in this review.

We investigated the effects of macronutrient intake on bone outcomes in human studies following the Preferred Reporting Items of Systematic Reviews and Meta-Analyses (PRISMA) statement [85]. Systematic research was conducted for manuscripts published up to 21 April 2023 in PubMed^®^/MEDLINE^®^ (https://www.ncbi.nlm.nih.gov/pubmed/ (accessed on 21 April 2023)). The manuscripts were limited to human studies written in English. We included studies that examined the association between macronutrients intake (including carbohydrate, protein, or fat) and bone-related outcomes. The search terms were combined with macronutrients or carbohydrate or protein or fat or fatty acid. All titles and abstracts were initially screened; after this stage, full-text manuscripts were retrieved and reviewed for final selection in line with the study eligibility criteria. The inclusion criteria were articles that analyzed the effects of macronutrients intake on bone outcomes (bone density, bone mineral density, bone mass, bone mineral content, bone turnover, bone markers, bone fracture, and bone health). Finally, we included meta-analyses of human studies, individual human studies addressed in the meta-analyses, and individual human studies not addressed in the meta-analyses. Manuscripts that did not meet the inclusion criteria above were excluded. Therefore, 11 meta-analyses and 127 individual human studies were included in this review. A flow diagram of the selection in this study is presented in Figure 1.

### Rationale for Not Conducting a Meta-Analysis

Due to the substantial heterogeneity in study designs, populations, interventions, and outcomes among the included studies, we deemed it inappropriate to conduct a meta-analysis, as it could potentially lead to misleading conclusions. However, we endeavored to provide a comprehensive synthesis of the available evidence to enable readers to draw informed conclusions.

## 3. Effects of Macronutrients on Bone Mineral Density, Bone Turnover Markers, and Bone Fracture

### 3.1. Carbohydrates

Table 1 shows the effects of carbohydrate on bone fracture. In summary, carbohydrate showed neutral effects on bone fracture.

#### 3.1.1. Bone Mineral Density and Bone Turnover Markers

We could not find any studies on the association between carbohydrate intake and BMD or BTMs.

#### 3.1.2. Bone Fracture

Mozaffari et al. (2020) [86] conducted a meta-analysis and a systematic review, as seen in Table 1. The meta-analysis of five observational studies [87,88,89,90,91] in individuals aged over 34 years showed no association between dietary carbohydrate consumption and bone fracture risk when comparing the highest with the lowest dietary carbohydrate consumption (overall RR = 1.24; 95% CI 0.84 to 1.84; *p* = 0.27; *I*^2^ = 57.7%; P_het_ = 0.05) [86].

### 3.2. Proteins

Table 2 shows the effects of protein on bone outcomes in meta-analyses of human studies. In summary, 17 meta-analyses of 57 human studies did not clearly show a positive effects of total protein on BMD, BTMs, and bone fracture. These three outcomes were not affected by different types of protein (total, animal, vegetable, soy, and milk basic protein (MBP)).

The effects of protein on bone outcomes in individual human studies are presented in Table 3, Table 4 and Table 5. As seen in Table 3, we extensively examined individual human studies including recent ones not included in the meta-analyses presented in Table 2. From the 96 studies (Table 3, Table 4 and Table 5), it is unclear whether total protein, animal protein, vegetable protein, soy protein, and MBP favorably influence BMD, BTMs, and bone fracture, even though an elevation in IGF-1 levels was observed in subjects with high total protein, soy protein, and MBP intake in seven studies. Total protein beneficially affected total hip BMD and total body BMD in six and three cross-sectional studies, respectively. Animal protein beneficially affecting LS BMD, and FN BMD was observed in two prospective studies. LS BMC was elevated in subjects who consumed soy protein and MBP in intervention studies. Moreover, MBP was associated with higher IGF-1 levels and lower urinary N-telopeptide of type 1 collagen (u-NTX) levels.

#### 3.2.1. Bone Mineral Density

In a meta-analysis by Darling et al. (2019) [94], dietary protein intake was not associated with FN BMD (*n* = 4786; r (fixed) = 0.07 (0.04 to 0.09); R^2^ = 0.005 (0.5%); *p* < 0.001; *I*^2^ = 26%; P_het_ = 0.15) in 17 studies [95,96,97,98,99,100,101,102,103,104,105,106,107,108,109,110,111] or lumbar spine (LS) BMD (*n* = 4257; r (random) = 0.09 (0.04 to 0.14); R^2^ = 0.008 (0.8%) *p* < 0.001; *I*^2^ = 58%; P_het_ = 0.001) in 17 studies [95,98,100,101,102,103,105,106,107,108,109,110,111,112,113,114,115].

Darling et al. (2019) [94] found no significant effect of protein supplementation on LS BMD (total *n* = 255, mean difference (MD) (fixed) = 0.04 (0.00 to 0.08; *p* = 0.07), *I*^2^ = 0%; P_het_ = 0.47) in a meta-analysis of RCTs [116,117] and no effect of protein supplementation on FN BMD (total *n* = 435; MD (random) = 0.01 (−0.03 to 0.05; *p* = 0.59); *I*^2^ = 68%; P_het_ = 0.04) in a meta-analysis of three RCTs [116,117,118].

In addition, Darling et al. (2019) [94] found no effects of milk basic protein on LS BMD in a meta-analysis of three RCTs [125,126,127] (MD (fixed) = 0.02 (0.00 to 0.08, *p* = 0.8)).

Shams-White et al. (2017) [132] conducted a systematic review and meta-analysis that included seven RCTs [117,133,134,135,136,140,161] and seven prospective cohort studies [148,162,163,164,165,166,167]. When they performed a meta-analysis of five RCTs [117,133,134,135,136], higher protein intake was more associated with LS BMD than lower protein intake (net percentage change = 0.52%; 95% CI 0.06% to 0.97%; *I*^2^ = 0%). No effect on total hip (TH) BMD (eight RCTs [117,118,133,134,135,136,137,161] and two cohort studies [148,165]) and FN BMD (eight RCTs [117,118,133,134,135,136,140,141] and five cohort studies [162,163,164,165,167]) was observed when comparing higher and lower protein intakes. It was found that higher protein intake could cause less total body (TB) BMD loss compared with lower protein intake (five RCTs [135,137,141,161,168] and two cohort studies [148,162]).

Darling et al. (2009) [158] reported a significant association between total protein consumption and LS BMD in a meta-analysis of six RCTs [125,126,143,145,159,160] but not in one of 18 cross-sectional studies [10,98,100,101,102,104,105,106,109,110,111,113,165,169,170,171,172,190].

#### 3.2.2. Bone Fracture

In a meta-analysis by Darling et al. (2019) [94] of three case–control studies [122,123,124], no association between total protein intake and fracture was found (odds ratio (OR) (random) = 0.69 (0.30 to 1.58; *p* = 0.38), *n* = 4 studies (4 data points as 1 study had independent subgroups which could both be entered) *I*^2^ = 65%; P_het_ = 0.03)).

In addition, Darling et al. (2019) [94] found no association between protein intake and the RR of osteoporotic fractures for total protein (RR (random) = 0.94; 0.72 to 1.23; *I*^2^ = 32%), animal protein (RR (random) = 0.98; 0.76 to 1.27; *I*^2^ = 46%), or vegetable protein (RR (fixed) = 0.97 (0.89 to 1.09; *I*^2^ = 15%)) in a meta-analysis of studies using total [91,129,130,131], animal [91,128,129,130], and vegetable proteins [91,129,130].

Shams-White et al. (2017) [132] observed that higher protein intake was not associated with hip fracture risk in a systematic review of nine cohort studies [91,120,121,128,129,131,148,183,185]; however, it was associated with overall fracture risk in a systematic review of four cohort studies [119,130,148,184], which had low quality and inconsistent results [132].

In a meta-analysis of four prospective studies [120,148,149,173] by Groenendijk et al. (2019) [191], dietary protein intake above the current recommended dietary allowance (RDA) of 0.8 g/kg of body weight/day was significantly associated with an 11% decreased hip fracture risk compared with a protein intake below it (pooled hazard ratio (HR): 0.89; 95% CI 0.84 to 0.94; *p* < 0.001).

A positive trend between higher protein intake and higher FN and TH BMD was observed [191]. Consistently, a meta-analysis by Wu et al. (2015) [192] of six prospective studies [120,121,129,131,148,193], as well as four using animal protein [91,128,130,194] and three on vegetable protein [184,194,195] with 407,104 subjects, reported that higher total protein intake was associated with an 11% reduction in the risk of hip fractures (RR = 0.89; 95% CI 0.82 to 0.97) [192].

Darling et al. (2009) [158] reported that no association between protein consumption and fracture risk was observed in four cohort studies.

#### 3.2.3. Bone Turnover Markers

Shams-White et al. (2018) [142] identified that higher protein intake was not associated with OC (from 10 RCTs [117,125,126,133,135,138,139,140,141,186]) and CTX (from 5 RCTs [117,133,137,139,141]) compared with lower protein intake.

### 3.3. Fat

The effects of fat on BMD, BTMs, and bone fracture in meta-analyses of human studies are presented in Table 6. In summary, the evidence for positive effects of total fat, monounsaturated fatty acid (MUFA), saturated fatty acid (SFA), total polyunsaturated fatty acid (PUFA), omega-3 fatty acid (N-3 PUFA), α-linolenic acid (ALA), and fish consumption on BMD, BTMs, and bone fracture outcomes was not sufficient based on five meta-analyses. Moreover, total PUFA including N-3 PUFA did not favorably influence these outcomes in five meta-analyses.

The effects of fat on BMD, BTMs, and bone fracture in individual human studies are presented in Table 7. In summary, eicosapentaenoic acid (EPA) and docosahexaenoic acid (DHA) had positive effects on OC according to two intervention studies. However, other positive effects on these outcomes were not shown in any type of fat intake.

#### 3.3.1. Bone Mineral Density

Dou et al. (2022) [196] performed a meta-analysis of six RCTs [197,198,199,200,201,202] that included 491 subjects aged 25 to 85 years. They found that N-3 PUFA significantly increased BMD (WMD = 0.005 g/cm^2^; 95% CI 0.00 to 0.01; *I*^2^ = 27.4%; P_het_ = 0.219).

Abdelhamid et al. (2019) [209] conducted meta-analyses that involved 7288 participants in 28 RCTs from 31 publications [197,199,200,201,202,203,210,211,212,213,228,229,230,231,232,233,234,235,236,237,238,239,240,241,242,243,244,245,246,247,248] to examine the effects of N-3 PUFA or total PUFA consumption on BMD outcomes by comparing high and low doses over more than 6 months. Higher N-3 PUFA intake was associated with a 2.6% increase in LS BMD (MD = 0.03 g/cm^2^, 95% CI −0.02 to 0.07; 463 participants) and a 4.1% increase in FN BMD compared with lower intake. However, no association between higher omega-3 intake and total bone mass was observed. In addition, no association between higher total PUFA intake and BMD was observed [209].

A meta-analysis by Lavado-García et al. (2018) [227] showed a positive association between dietary N-3 PUFA intake and BMD in normal and osteopenic Spanish women aged 20–79 years old. Moreover, dietary intake of DHA was significantly associated with LS BMD in normal women. However, no association between dietary N-3 PUFA consumption and BMD at LS was observed in osteopenic or osteoporotic women [227].

#### 3.3.2. Bone Fracture

A meta-analysis of observational studies (four prospective studies [215,216,217,218] and two case–control studies [219,220]) by Sadeghi et al. (2019) [214] showed significant inverse associations between fish intake (pooled effect size = 0.88; 95% CI 0.79 to 0.98; *p* = 0.02) or dietary N-3 PUFA intake (pooled effect size = 0.89, 95% CI 0.80 to 0.99, *p* = 0.02) and hip fracture risks [214].

Another meta-analysis of six observational studies [88,89,90,222,224,225] by Mozaffari et al. (2018) [223] showed that risk of hip fractures had a significant positive association with the intake of SFA (pooled effect size = 1.79; 95% CI 1.05 to 3.03; *p* = 0.03) or animal-derived MUFA (pooled effect size = 2.29; 95% CI 1.50 to 3.50; *p* < 0.0001). However, no significant association was found between total dietary fat intake and risk of fracture [223].

#### 3.3.3. Bone Turnover Markers

Dou et al. (2022) [196] performed four meta-analyses of BTM outcomes from 10 RCTs [197,198,199,200,201,202]. A meta-analysis of seven RCTs [197,200,203,204,205,206,207] showed no association between N-3 PUFA intake and bone-specific alkaline phosphatase (BSAP) (WMD = −0.24; 95% CI −0.86 to 0.39; *I*^2^ = 47.4%; P_het_ = 0.076) [196]. In a meta-analysis of five RCTs [197,200,201,203,208] by Dou et al. (2022) [196], N-3 PUFA intake was not associated with OC (WMD = −0.63; 95% CI −1.84 to 0.57; *I*^2^ = 43.9%; P_het_ = 0.129). Moreover, a meta-analysis of three RCTs [197,203,205] by the same authors [196] found no association between N-3 PUFA intake and NTX (WMD = −1.74; 95% CI −3.97 to 0.48; *I*^2^ = 65.8%; P_het_ = 0.054). However, the intake of N-3 PUFA was found to be associated with lower CTX levels (WMD = −0.37; 95% CI −0.73 to −0.01; *I*^2^ = 94.8%; P_het_ = 0.000) in a meta-analysis of four RCTs [201,202,205,206] by Dou et al. (2022) [196].

From a meta-analysis of eight RCTs, Shen et al. (2017) [226] reported that N-3 PUFA had an effect on BTMs in postmenopausal women [197,200,201,203,204,206,208,213]. N-3 PUFA significantly reduced serum OC concentrations (WMD = −0.86 ng/mL; 95% CI −1.68 to −0.04; *p* = 0.040) compared with the control group, while changes in BSAP (needed for bone calcification) and CTX were not observed [226].

## 4. Discussion

The objective of this review was to clarify the effects of macronutrients and/or carbohydrate and/or fat and/or protein on bone health in elderly people with a focus on human studies.

Herein, we found neutral effects of carbohydrate consumption on bone fracture. A meta-analysis of three case–control and two prospective studies showed that carbohydrate consumption did not significantly increase nor decrease fracture risks [86]. Similar results were found by Benetou et al. (2011) [93], who observed no association between carbohydrate intake and the prevalence of hip fracture in a European Prospective Investigation into Cancer and Nutrition (EPIC) cohort study [93]. Inconsistently, Huang et al. (1996) [92] showed an association between increased carbohydrate intake and a lower risk of hip fracture in 2513 white women aged over 45 years [92] based on prospective data from National Health and Nutrition Examination Survey (NHANES) follow-up studies.

The present study did not find an association between carbohydrate intake and BMD or/and BTMs in the human studies analyzed. Gao et al. (2022) [249] recently observed that a higher proportion of energy from carbohydrate was associated with a lower BMD T-score and a higher risk of bone loss among 4447 adults aged over 20 years in NHANES data. Moreover, Mazidi et al. (2018) [250] showed that diets high in carbohydrates, sugar, total fat, and saturated fat were associated with a lower BMD in the total femur, femoral neck, trochanter, and intertrochanter, whereas diets rich in vitamins, minerals, fiber, PUFAs, and MUFAs were associated with a higher BMD. Even though these studies [249,250] showed some negative effects of carbohydrate intake on BMD or BTMs, they did not sufficiently support the association between these factors. Therefore, many more human studies are required to clarify the association between carbohydrates and bone outcomes.

Taking into consideration the five meta-analyses [196,209,214,223,226] addressed in this study, positive effects of total fat, MUFA, SFA, PUFA, N-3 PUFA, ALA, and fish intake on BMD, BTMs, and bone fractures were not observed. In addition, no effects on these outcomes were found in any type of fat intake in a review of individual human studies. However, two intervention studies [201,208] observed favorable effects of EPA and DHA intake on OC levels. In an intervention of 40 patients with osteoporosis [208], OC levels were higher in the group consuming a mixture of evening primrose and fish oil compared to the evening primrose oil-only group. Omega-3 supplementation with 24 weeks of exercise increased OC levels [201].

In the present study, we could not find the apparent association between FN BMD and N-3 PUFA after reviewing five human studies [199,201,202,212,227]. Dodin et al. (2005) [199] observed BMD changes in postmenopausal women who consumed ALA for 12 months compared with the placebo group, but changes in LS BMD and FN BMD were not observed between these two groups. In other interventions [202,212], 40 women supplemented with DHA for 12 months showed no differences in LS, TH, and FN BMD compared to the control [202]. The LS and FN BMD of subjects who received high- or low-dose omega-3 fish oil were not significantly changed [212]. Inconsistently, a cross-sectional study by Lavado-García et al. (2018) [227] showed a positive association between ALA, EPA, DHA and FN BMD in all (premenopausal and postmenopausal women) and premenopausal women. Beneficial effects on LS BMD (L2-L4) were also shown with EPA and DHA in all (premenopausal and postmenopausal women) and premenopausal women.

Rajaram et al. (2017) [251] observed that an alteration in the ratio of N-6:N-3 PUFA from 10:1 to 2:1 for 8 weeks did not affect BTMs and PPARγ in an 8-week crossover trial with a 4-week washout period [251]. PPARγ is known to be a mediator in the adipogenesis of glucose and fat metabolism [252,253]. Mesenchymal stem cells (MSCs) possess the remarkable ability to differentiate into various lineages, notably adipocytes (fat cells) and osteoblasts (cells that form bone). A pivotal player in this differentiation process is PPARγ. When activated, it fosters adipogenesis, simultaneously downregulating osteoblastic genes and upregulating adipogenic genes. This shift in gene expression propels MSCs toward adipocyte differentiation, often at the detriment of osteoblastogenesis, leading to diminished bone formation [252]. Furthermore, PPARγ extends directly to osteoblasts. Its activation can stymie the proliferation and functionality of osteoblasts, further curtailing bone formation. Osteoclasts, the cells tasked with bone resorption, also interact with PPARγ, albeit in a more intricate manner. Research indicates that PPARγ might impede osteoclast differentiation and activity, which would theoretically reduce bone resorption. Nevertheless, the overarching impact of PPARγ on bone predominantly leans toward bone degradation, which is largely attributed to its modulation of osteoblast activity and the adipogenesis–osteoblastogenesis equilibrium [252]. This intricate interplay between PPARγ and bone metabolism becomes evident when examining thiazolidinediones (TZDs), which is a drug class prescribed for type 2 diabetes. As PPARγ agonists, TZDs enhance insulin sensitivity. However, they come with a caveat: they have been linked with diminished bone density and a heightened risk of fractures in certain individuals. This adverse effect is postulated to stem, at least partially, from PPARγ’s modulation of bone metabolism [253]. To sum up, while PPARγ is instrumental in regulating fat and glucose metabolism, its activation also exerts profound effects on bone metabolism. This primarily manifests as a tilt in the balance favoring fat cell formation over bone cell formation within the bone marrow milieu coupled with a direct impact on the activity of bone-forming cells.

In this study, the positive effects of total protein on BMD, BTMs, and bone fracture were not clearly shown based on 17 meta-analyses of 57 human studies. Moreover, seven individual studies [73,117,118,140,143,150,187] reported an increase in IGF-1 in subjects who consumed higher intakes of total, soy, and milk basic proteins.

We observed higher TH BMD (in six cross-sectional studies [95,97,101,113,115,177]) and higher TB BMD (in three cross-sectional studies [106,109,115]) after the consumption of total protein. In addition, two prospective studies [163,179] reported evidence of increased animal protein benefiting LS BMD and FN BMD. Human studies [254,255] showed the effects of protein intake on BMD. Groenendijk et al. (2023) [254] showed that total protein supplementation was associated with higher TB BMD and LS BMD along with animal protein supplementation [254]. Steell et al. (2019) [255] also showed a positive association between protein intake and BMD in a cross-sectional study of 70,215 men and women.

IGF-1 generated from body tissues, including bone, is a polypeptide hormone that regulates bone-related cells [256,257]; it stimulates the absorption of phosphate in the plasma membrane of osteoblastic cell lines, which contributes to bone formation [258,259]. The imbalance of IGF-1 in bone tissues caused by aging [260,261], obesity [262,263], or other factors can result in the onset of the disease osteoporosis [264]; decreased levels of this hormone induced by low protein intake could result in an elevated risk of osteoporosis and bone fracture [265,266].

We found that MBP intake was associated with increased IGF-1 (in two studies [117,118]) and decreased urinary NTX (in three studies [125,126,186]). However, Fuglsang-Nielsen et al. (2022) [267] showed no effects of whey protein supplementation for 12 weeks on plasma P1NP and CTX in 64 prediabetic subjects with abdominal obesity. Protein intake is linked to the stimulation of IGF-1, which helps bone growth [268,269].

The strengths of this review are that we attempted to extensively examine human studies, including recent studies, as much as possible. This work provides an update on recent evidence surrounding the influence of each macronutrient (carbohydrates, proteins, and fats) on bone outcomes based on human studies.

Nevertheless, this review has limitations. We could not find human studies which investigated the effects of carbohydrates on BMD and BTMs; this review only focused on the effects of macronutrients on bone health. Therefore, future studies should include intervention studies examining the association between carbohydrates and BMD and BTMs. Research is needed to clarify how the interaction of macronutrients and micronutrients affects bone health.

## 5. Conclusions

In conclusion, carbohydrate consumption appeared to have neutral effects on bone fracture. The beneficial influences of total protein, animal protein, vegetable protein, soy protein, and MBP on bone outcomes were unclear based on inconsistent study findings. The consumption of omega-3 fatty acids appeared to be associated with osteocalcin.

In future, well-designed, long-term human intervention studies are required to examine the association between nutrients and bone health in elderly people. Moreover, epidemiological or/and intervention studies investigating the influence of carbohydrates on bone health should be performed.

## Figures and Tables

**Table 1:** The effects of carbohydrates on bone fracture outcomes in human studies.

Ref	Nutrient Type	Description	Study Type; N of Subjects	Follow-Up Period and Age Range or Mean Age	Bone Fracture Outcomes
Mozaffari et al., 2020 [86]	CHO	Meta-analysis of five studies [87,88,89,90,91]	Observational; 38,828 subjects	3–7.6 years ≥34 years	↔ fracture risk in high-carbohydrate-intake group (overall RR (random) = 1.24; 95% CI 0.84 to 1.84; *p* = 0.27; *I*^2^ = 57.7%; P_het_ = 0.05) (vs. low)
Xu et al., 2009 [87]			Case–control; 418 subjects	N/A 61 years	↔ fracture risk in high-intake group (vs. low)
Kato et al., 2000 [88]			Prospective; 4884 subjects	7.6 years 34–65 years	↔ fracture risk in high-intake group (vs. low)
Michaelson et al., 1995 [89]			Case–control; 1140 subjects	N/A 67 years	↔ fracture risk in high-intake group (vs. low)
Ramirez et al., 2007 [90]			Case–control; 334 subjects	N/A 72 years	↔ fracture risk in high-intake group (vs. low)
Munger et al., 1999 [91]			Prospective; 32,050 subjects	3 years 55–69 years	↔ fracture risk in high-intake group (vs. low)
Huang et al., 1996 [92]			Prospective; 2513 subjects	13.4 years 45–77 years	↓ fracture risk by 20% in high-intake group (vs. low)
Benetou et al., 2011 [93]			Prospective; 29,122 subjects	8 years 60–86 years	↔ fracture risk in high-intake group (vs. low)

CHO, carbohydrate; CI, confidence interval; het, heterogeneity; HR, hazard ratio; N, number; N/A, not available; OR, odds ratio; RR, relative risk; ↓, decrease; ↔, no effect.

**Table 2:** The effects of protein on bone outcomes in meta-analyses of human studies.

Ref	Nutrient Type	Description	Studies	Study Type; N of Subjects	Follow-Up Period Age Range or Mean Age	BMD and/or Bone Fracture and/or BTM Outcomes
Darling et al., 2019 [94]	Total protein	Four meta-analyses of BMD outcomes	19 studies [95,96,97,98,99,100,101,102,103,104,105,106,107,108,109,110,111]	Cross-sectional; 4786 subjects	N/A 20–89 years	↔ FN BMD with total protein intake (r (fixed) = 0.07; 95% CI 0.04 to 0.09; R^2^ = 0.005 (0.5%); *p* < 0.0001; *I*^2^ = 26%; P_het_ = 0.15)
			18 studies [95,97,98,100,101,102,103,105,106,107,108,109,110,111,112,113,114,115]	Cross-sectional; 4257 subjects	N/A 20–89 years	↔ LS BMD with total protein intake (r (random) = 0.09; 95% CI 0.04 to 0.14; R^2^ = 0.008 (0.8%); *p* < 0.001; *I*^2^ = 58%; P_het_ = 0.001)
			Two studies [116,117]	RCT; 255 subjects	7–18 months ≥60 years	↔ LS BMD with total protein intake (MD (fixed) = 0.04; 95% CI 0.00 to 0.08; *I*^2^ = 0.0%; P_het_ = 0.47)
			Three studies [116,117,118]	RCT; 435 subjects	7–24 months ≥60 years	↔ FN BMD with total protein intake (MD (random) = 0.01; 95% CI −0.03 to 0.05; *I*^2^ = 68%; P_het_ = 0.04)
		Two meta-analyses of bone fracture outcomes	Three studies [119,120,121]	Prospective; 9263 subjects	12–17 years (14) 20–62 years	↔ HR for all fractures with total protein intake (HR (random) = 0.82; 95% CI 0.59 to 1.14; *p* = 0.24; *I*^2^ = 35%; P_het_ = 0.19)
			Three studies [122,123,124]	Case–control; 3164 subjects	N/A 50–103 years	↔ OR of fracture (OR (random) = 0.69; 95% CI 0.30 to 1.58; *p* = 0.38; *I*^2^ = 65%; P_het_ = 0.03)
	MBP	A meta-analysis of BMD outcomes	Three studies [125,126,127]	RCT; 115 subjects	6–8 months 30.5 years	↔ LS BMD (MD (fixed) = 0.02; 95% CI 0.00 to 0.04; *p* = 0.08; *I*^2^ = 0.0%; P_het_ = 0.87)
	Animal protein	Three meta-analyses of bone fracture outcomes	Four studies [91,128,129,130]	Prospective; 193,954 subjects	3–12 years (9.6) 30–69 years	↔ all low-trauma fractures (RR (random) = 0.98; 95% CI 0.76 to 1.27; *p* = 0.87; *I*^2^ = 46% P_het_ = 0.13)
	Vegetable protein		Three studies [91,129,130]	Prospective; 154,167 subjects	3–12 years (9) 30–69 years	↔ all low-trauma fractures (RR (fixed) = 0.97; 95% CI 0.89 to 1.09; *p* = 0.61; *I*^2^ = 15%; P_het_ = 0.31)
	Total protein		Four studies [91,129,130,131]	Prospective; 156,416 subjects	3–13.9 years (10.2) 30–69 years	↔ all low-trauma fractures (RR = 0.94; 95% CI 0.72 to 1.23; *p* = 0.55; *I*^2^ = 32%; P_het_ = 0.31)
Shams-White et al., 2017 [132]	Total Protein	Three meta-analyses of BMD outcomes	Five studies [117,133,134,135,136]	RCT; 989 subjects	12–24 months (18) ≥40 years	↑ LS BMD with higher protein (net percentage change = 0.52%; 95% CI 0.06 to 0.97; *I*^2^ = 0.0%; P_het_ = 0.579) (vs. lower)
			Six studies [117,118,133,134,135,136]	RCT; 1172 subjects	12–24 months (22.8) ≥40 years	↔ FN BMD on higher protein intake (pooled mean percentage change = −0.14%; 95% CI −0.60 to 0.32; *I*^2^ = 0.0%; P_het_ = 0.952) (vs. lower)
			Seven studies [117,118,133,134,135,136,137]	RCT; 1208 subjects	12–24 months (18) ≥40 years	↔ TH BMD on higher protein intake (pooled net percentage change = 0.30%; 95% CI −0.02 to 0.62; *I*^2^ = 0.0%; P_het_ = 0.539) (vs. lower)
		Two meta-analyses of BTM outcomes	Eight studies [117,125,133,135,138,139,140,141]	RCT; 494 subjects	6–24 months (12.8) 40–92 years	↔ OC on higher protein intakes (pooled net change: 0.06 ng/mL; 95% CI −0.49 to 0.60; *I*^2^ = 27.2%; P_het_ = 0.211) (vs. lower)
			Five studies [117,133,137,139,141]	RCT; 370 subjects	12–24 months (15.6) 40–92 years	↔ CTX in higher protein intake (pooled net change = 47.72 ng/L; 95% CI −27.34 to 122.78; *I*^2^ = 61.3%; P_het_ = 0.035) (vs. lower)
Shams-White et al., 2018 [142]	Isoflavone -rich soy protein vs. animal protein	Three meta-analyses of BMD outcomes	Four studies [143,144,145,146]	RCT; 393 subjects	12–24 months (15) 66 years	↔ LS BMD (pooled mean percentage change = 0.24%; 95% CI −0.80 to 1.28; *I*^2^ = 0.0%)
			Three studies [144,145,146]	RCT; 331 subjects	12–24 months (16) 67.8 years	↔ FN BMD (pooled mean percentage change = 0.13%; 95% CI = −0.94 to 1.21; *I*^2^ = 0.0%)
			Three studies [143,144,146]	RCT; 218 subjects	12–24 months (16) 63.7 years	↔ TB BMD (pooled mean percentage change = −0.24%; 95% CI −0.81 to 0.33; *I*^2^ = 0.0%)
Wallace and Frankenfeld et al., 2017 [147]	Total protein	A meta-analysis of bone fracture outcomes	Five studies [91,120,131,148,149]	Prospective; 289,707 subjects	1–22 years (12.4) 20–79 years	↓ hip fractures in higher protein intake (SMD = 0.84%; 95% CI 0.73 to 0.95; *I*^2^ = 36.8%; P_het_ = 0.161) (vs. low)
		Two meta-analyses of BTM outcomes	13 studies [73,82,117,150,151,152,153,154,155,156]	RCT; 509 subjects	4 days to 9 weeks 20–75 years	↑ urinary Ca excretion with protein intake (SMD = 0.48; 95% CI = 0.30 to 0.66; *I*^2^ = 28.3%; P_het_ = 0.167)
			Seven studies [73,125,150,152,155,157]	RCT; 243 subjects	4 days to 9 weeks 20–75 years	↔ u-NTX with protein intake (SMD = −0.18; 95% CI −0.99 to 0.26; *I*^2^ = 66.3%; P_het_ = 0.007)
Darling et al., 2009 [158]	Total protein	Three meta-analyses of BMD outcomes	Three studies [116,125,126]	RCT; 110 subjects	6–7 months (6.3) 51.3 years	↔ LS BMD with protein supplementation (WMD (fixed) = 0.02; 95% CI 0.00 to 0.04; *p* = 0.04; *I*^2^ = 0.0%; P_het_ = 0.62)
	Soy protein		Three studies [145,159,160]	RCT; 264 subjects	6–12 months (8) 44–75 years	↔ LS BMD with soy protein supplementation (WMD (fixed) = 0.01; 95% CI −0.05 to 0.06; *p* = 0.86; *I*^2^ = 54.1%; P_het_ = 0.11)
	MBP		Two studies [125,126]	RCT; 62 subjects	6 months 35.9 years	↔ LS BMD with MBP supplementation (WMD (fixed) = 0.02; 95% CI 0.00 to 0.04; *p* = 0.07; *I*^2^ = 0.0%; P_het_ = 0.85)
	Total Protein	Three meta-analyses of bone fracture outcomes	Three studies [91,129,131]	Prospective; 120,199 subjects	3–13.9 years (9.6) 30–74 years	↔ fracture risk in the highest quintile of total protein intake (RR (random) = 0.75; 95% CI 0.47 to 1.21; *p* = 0.23; *I*^2^ = 20.4%; P_het_ = 0.28) (vs. lowest)
	Animal protein		Three studies [91,128,129]	Prospective; 157,737 subjects	3–12 years (8.8) 30–69 years	↔ fracture risk in the highest quintile of animal protein intake (RR (random) = 0.83; 95% CI = 0.54 to 1.30; *p* = 0.42; *I*^2^ = 48.3%; P_het_ = 0.14) (vs. lowest)
	Vegetable protein		Two studies [91,129]	Prospective; 117,950 subjects	3–12 years (7.5) 30–69 years	↔ fracture risk in the highest quintile of vegetable protein intake (RR (random) = 1.21; 95% CI 0.82 to 1.79; *I*^2^ = 2.0%; *p* = 0.34; P_het_ = 0.31) (vs. lowest)

BMD, bone mineral density; BTM, bone turnover marker; Ca, calcium; CI, confidence interval; CTX, C-terminal telopeptide cross-link of type 1 collagen; FN, femoral neck; HR, hazard ratio; het, heterogeneity; LS, lumbar spine; MBP, milk basic protein; MD, mean difference; N, number; N/A, not available; OC, osteocalcin; OR, odds ratio; RCT, randomized controlled trial; RR, relative risk; SMD, standardized mean difference; TB, total body; TH, total hip; u-NTX, urinary N-telopeptide of type 1 collagen; WMD, weighted mean difference; ↑, increase; ↓, decrease; ↔, no effect.

**Table 3:** The effects of proteins on bone mineral density outcomes in individual human studies.

Nutrient Type	Ref	Study Type	N of Subjects Study Design	Follow-Up Period and Age	BMD Outcomes
Total protein	Kyriazopoulos et al., 2006 [10]	Cross- sectional	300 healthy Caucasian men Four categories of protein intake (g/week): Group 1: 0–84; Group 2: 126–168; Group 3: 210–252; Group 4: 294–420	N/A 18–30 years (22.58 ± 3.34)	↔ distal radius BMD or BMC with protein intake
Total protein	Alissa et al., 2014 [95]	Cross- sectional	300 postmenopausal Saudi women	N/A 46–88 years (59.9 ± 0.5)	↔ LS BMD with energy-adjusted protein ↑ FN BMD (r = 0.182), TH BMD (r = 0.244) with energy-adjusted protein
	Chan et al., 2009 [96]	Cross- sectional	441 premenopausal women	N/A 20–35 years	↓ TH BMD (r = −0.103) with dietary protein ↔ FN BMD and LS BMD with dietary protein
	Coin et al., 2008 [97]	Cross- sectional	352 elderly outpatients	N/A Men: 73.9 ± 5.6 years Women: 73.5 ± 5.3 years	↑ TH BMD (R^2^ = 0.06) and troch BMD (R^2^ = 0.08) in men ↔ FN BMD in men
	Chiu et al., 1997 [98]	Cross- sectional	258 postmenopausal Taiwanese women Exposure: protein intake (% of E)	N/A 40–87 years (60.79 ± 9.23)	↑ LS BMD (β = 0.039) with energy intake from protein ↔ FN BMD (β = 0.012) with energy intake from protein ↓ LS osteopenia by 49% after multivariate adjustment ↔ FN osteopenia after multivariate adjustment
	Guun et al., 2014 [99]	Cross- sectional	142 healthy postmenopausal women	N/A 50–70 years	↑ FN BMD after adjustment for energy values (r = 0.19)
	Cooper et al., 1996 [100]	Cross- sectional	290 pre- and postmenopausal women	N/A Premenopausal women: 39 years Postmenopausal women: 68 years	↑ femoral troch BMD (r = 0.35), FN BMD (r = 0.27), and distal radius BMD (r = 0.28) in premenopausal women after multivariate adjustment ↔ LS BMD, midradius BMD, and femoral shaft BMD after multivariate adjustment ↔ LS BMD, femoral troch BMD, FN BMD, distal radius BMD, midradius BMD, and femoral shaft BMD in postmenopausal women after multivariate adjustment
	Henderson et al., 1995 [101]	Cross- sectional	115 healthy, sexually mature Caucasian women	N/A 18 years	↔ LS BMD, femoral shaft BMD, and distal tibia and fibula BMD after multivariate adjustment ↑ FN BMD (r = 0.22), troch BMD (r = 0.27), intertrochanter BMD (r = 0.19), and TH BMD (r = 0.21) after multivariate adjustment
Soy protein	Ho et al., 2003 [102]	Cross- sectional	454 healthy Chinese women within the first 12 years of menopause	N/A 48–62 years (55.1 ± 3.57)	↔ LS BMD, FN BMD, troch BMD, intertrochanter BMD, TH BMD, and TB BMD after multivariate adjustment
Total protein	Kumar et al., 2010 [103]	Cross- sectional	225 healthy women	N/A 20–69 years (40.5 ± 12.7)	↑ LS BMD after multivariate adjustment (r = 0.224) ↔ FN BMD and Ward BMD after multivariate adjustment
Total protein	Jaime et al., 2006 [104]	Cross- sectional	277 Brazilian black and white men	N/A >50 years (white, 62.6 ± 8.14; black, 59.7 ± 5.63)	↔ FN BMD in the white men (r = 0.055) after adjusting for energy intake ↑ FN BMD in the black men (r = 0.359) after adjusting for energy intake ↔ FN BMD in the white men (β = 0.00058) and black men (β = 0.00192) after adjusting for energy intake
Total protein	Lau et al., 1998 [105]	Cross- sectional	76 vegetarian Chinese women	N/A 70–89 years (79.1 ± 5.2)	↔ LS BMD, FN BMD, intertrochanter BMD, and Ward BMD after multivariate adjustment
Total protein	Michaëlsson et al., 1995 [106]	Cross- sectional	175 Caucasian women	N/A 28–74 years	↔ TB BMD and LS BMD with nutrients from dietary records after multivariate adjustment ↑ FN BMD with nutrients from dietary records after multivariate adjustment (β = 0.0028) ↑ TB BMD with nutrients estimated from FFQ after multivariate adjustment (β = 0.0020) ↔ LS BMD and FN BMD with nutrients estimated from FFQ after multivariate adjustment
Total protein	New et al., 1997 [107]	Cross- sectional	994 healthy premenopausal women	N/A 45–49 years (47.1 ± 1.43)	↔ LS BMD, FN BMD, femoral troch BMD, and femoral Ward BMD after multivariate adjustment
Total protein	Orozco López et al., 1998 [108]	Cross- sectional	76 premenopausal women Mean protein intake (g/day): Total protein: 73.4; Animal protein: 49.7; Vegetable protein: 23.7.	N/A 42 years	↔ LS BMD, FN BMD, troch BMD, intertrochanter BMD, and Ward BMD with protein intake
Total protein	Rapuri et al., 2003 [109]	Cross- sectional and Prospective	473 postmenopausal women Dietary protein intake (% of E) Q1: 13.1 ± 0.12; Q2: 15.1 ± 0.11; Q3: 16.7 ± 0.12; Q4: 19.8 ± 0.12.	N/A 65–77 years	Cross-sectional analysis: ↑ LS BMD in Q4 of protein intake (vs. Q2, Q3) ↑ midradius BMD and TB BMD in Q4 of protein intake (vs. Q2) ↔ FN BMD, troch BMD, and TH BMD ↑ LS BMD with protein in Q3 and Q4 of Ca intake (vs. Q1 Ca intake) ↔ TB BMD with protein intake in Q3 and Q4 of Ca intake (vs. Q1 intake) ↔ midradius BMD, troch BMD, and TH BMD with protein intake and Ca intake Prospective analysis: ↔ TH BMD, FN BMD, troch BMD, Ward, TB BMD, and radius BMD with protein intake
Total protein	Teegarden et al., 1998 [110]	Cross- sectional	215 white women	N/A 18–31 years (23.8 ± 3.6)	↑ radius BMD and LS BMD
Total protein	Wang et al., 1997 [111]	Cross- sectional	125 Mexican American Caucasian women	N/A 59–84 years (68.0 ± 5.1)	↔ FN BMD and LS BMD
Soy protein	Horiuchi et al., 2000 [112]	Cross- sectional	85 postmenopausal women	N/A 52–83 years (66.9 ± 7.4)	↔ LS BMD after multivariate adjustment
Total protein	Quintas et al., 2003 [113]	Cross- sectional	164 women	N/A Control: 16.2 ± 1.0 years Dancers: 16.2 ± 2.0 years Basketballers: 17.2 ± 2.1 years Skiers: 17.1 ± 2.9 years	↑ LS BMD (r = 0.31726) and right hip BMD (r = 0.3005) after multivariate adjustment
Total protein	Thorpe et al., 2008 [114]	Cross- sectional	161 postmenopausal women	N/A 67.9 ± 7.4 years	↑ LS areal BMD with a direct effect of protein intake ↑ TH areal BMD on protein intake
Total protein	Whiting et al., 2002 [115]	Cross- sectional	57 men	N/A 39–42 years (39.6 ± 0.6)	↑ TB BMD (r = 0.383), hip BMD (r = 0.322), LS BMD (r = 0.419), and TB BMD (β = 0.00193; SE = 0.00065; t = 2.96) after multivariate adjustment
Total protein	Tkatch et al., 1992 [116]	Parallel RCT	48 elderly men and women Intervention (g/day): Protein: 20.4; control: 0	7 months ≥60 years (82)	↔ FN BMD, femoral shaft BMD, and LS BMD between groups ↑ femoral shaft BMD within the protein group
MBP	Kerstetter et al., 2015 [117]	Parallel RCT: double blind	208 men and women Intervention (g/day): Whey protein: 45 of whey protein Control: 0 All subjects: 400 IU vitamin D	18 months Men: ≥70 years Women: ≥60 years	↔ LS BMD, TH BMD, and FN BMD
MBP	Zhu et al., 2011 [118]	Parallel RCT: double blind	186 healthy ambulant postmenopausal women Protein intake (g/day): Protein: 30 (whey protein + skim milk); placebo: 2.1 (skim milk)	2 years 70–80 years (74.3 ± 2.7)	↔ TH BMD between groups ↔ FN BMD between groups and within groups
MBP	Aoe et al., 2005 [125]	Parallel RCT: double blind	27 healthy menopausal women Protein intake (mg/day): MBP group: 40; placebo group: 0	6 months 50.5 ± 3.0 years	↑ LS BMD in the MBP group (vs. placebo)
MBP	Uenishi et al., 2007 [126]	Parallel RCT: double blind	35 healthy young women Protein intake (mg/day): MBP: 40; placebo: 0	6 months 21.3 ± 1.2 years	↑ LS BMD gain in the MBP group (vs. placebo)
MBP	Zou et al., 2009 [127]	Parallel RCT	81 healthy young women Intervention (/day): MBP (40 mg of milk) group: 250 mL whole milk + 40 mg of MBP Whole-milk group: 250 mL Whole-milk control group: N/A	8 months 19.6 ± 0.6 years	↑ TB BMD within all groups ↔ LS BMD and left forearm BMD
Total protein	Jesudason et al., 2013 [133]	Parallel RCT	136 postmenopausal women Protein intake (g/day) High protein (HP): >90 High normal protein (HNP): <80	24 months 40–70 years (HP: 59.5 ± 0.4; HNP: 59.4 ± 0.4)	↔ L2–L4 BMD, distal forearm BMD, TH BMD, and FN BMD in the HP group (time, diet, diet × time vs. the HNP group)
MBP	Kukuljan et al., 2009 [134]	Parallel RCT	175 healthy men Protein intake (g/day): Milk: 13.2; Control: 0	12 months 50–79 years (MBP: 61.7 ± 7.7; control: 59.9 ± 7.4)	↑ TH BMD within the milk group ↔ FN BMD, LS BMD, TH BMD, and troch BMD with milk intake after adjusting for changes in weight
Total protein	Sukumar et al., 2011 [135]	Parallel RCT	47 healthy overweight/obese postmenopausal women Protein intake (% of E): HP: 30; NP: 18	1 year 58 ± 4 years	↑ LS BMD in the HP group (vs. NP) ↔ TB BMD, FN BMD, TH BMD, and BMC
Total protein	Tirosh et al., 2015 [136]	Parallel RCT	424 healthy adults Protein intake (% kcal/day): High protein: 25 (35% and 55% carbohydrate group) Average protein: 15 (45% and 65% carbohydrate group)	24 months 51.8 ± 8.9 years	↔ LS BMD and FN BMD
MBP	Flodin et al., 2014 [137]	Parallel RCT	67 patients with a recent hip fracture Intervention (/day): Bisphosphonates + nutritional supplementation (BN): 40 g of MBP + 5 mg of risedronate Bisphosphonates (B): 0 g of MBP + 5 mg of risedronate Controls (C): placebo All subjects: 1000 mg of Ca + 800 IU vitamin D_3_	1 year >60 years (79 ± 9)	↔ TB BMD, TH BMD
MBP	Holm et al., 2008 [139]	Parallel RCT: double blind	29 healthy, early postmenopausal women Intervention (/day): Nutrient (NUT): 10 g of whey protein, 31 g of carbohydrate, 1 g of fat, 5.0 μg of vitamin D, and 250 mg of Ca Control (C): 6 g of carbohydrate and 12 mg of Ca	24 weeks Nut: 55 ± 1 years C: 55 ± 1 years	↑ LS BMD within groups ↔ FN BMD, TB BMD within groups
MBP	Schürch et al., 1998 [140]	Parallel RCT: double blind	82 orthopedic patients with recent hip fracture Intervention (g/day): Protein: 20 milk protein (5 days/week); Control: 0	12 months >60 years (protein: 81.1 ± 7.4; control: 80.2 ± 7.4)	↔ LS BMD, FN BMD, troch BMD, femoral shaft BMD, and TB BMC between groups ↑proximal femur BMD in the protein group (vs. control)
MBP	Tengstrand et al., 2007 [141]	Parallel RCT	52 lean, postmenopausal patients with recent FN fracture Intervention (g/day): Nutrition (PR) and combined therapy (PR/N): 20 Controls (C): 0 All subjects: 1 g of Ca + 800 IE vitamin D	12 months 70–92 years (83 ± 5)	↑ TB BMD within the PR group at month 6 and 12 ↔ FN BMD within the PR group
Soy protein	Arjmandi et al., 2005 [143]	Parallel RCT: double blind	62 postmenopausal women Intervention (/day): Soy: 25 g of soy protein + 60 mg of isoflavones Control: 25 g of non-soy protein	1 year <65 years (soy: 53 ± 6; control: 56 ± 5)	↔ LS BMD, TH BMD, TB BMD, TB BMC, LS BMC, and TH BMC in the soy group (vs. control)
Soy protein	Kenny et al., 2009 [144]	Parallel RCT: double blind	97 healthy ambulatory postmenopausal women Intervention (/day): Soy protein placebo (SPI−), soy protein isoflavone (SPI+): 18 g of soy protein Control protein placebo, control protein isoflavone: 18 g of milk and egg white protein Co-intervention (/day): SPI+: 35 mg of isoflavone All subjects: if not achieving 1200–1500 mg of Ca via diet, they were administered 315 mg of Ca and 200 IU vitamin D	1 year >60 years (73.1 ± 5.9)	↔ TB BMD, FN BMD, and LS BMD between groups
Soy protein	Kreijkamp et al., 2004 [145]	Parallel RCT: double blind	175 healthy postmenopausal women Intervention (g/day): Soy protein + isoflavones (SPI+): 25.6 isoflavone-rich soy protein Placebo: 25.6 milk protein	1 year 60–75 years (SPI+, 66.5 ± 4.7; placebo, 66.7 ± 4.8)	↔ FN BMD, LS BMD, and TH BMD in the SPI+ group (vs. placebo)
Soy protein and MBP	Vupadhyayula et al., 2009 [146]	Parallel RCT: double blind	157 healthy postmenopausal women Intervention (g/day): Soy protein: 25 of soy protein isolate; soy protein + isoflavone: 25 of soy protein isolate + 90 mg of isoflavone; milk protein: 25 of casein and whey	2 years Soy protein: 63.6 ± 0.6 years Soy protein + isoflavone: 63.4 ± 0.6 years Milk protein: 63.8 ± 0.5 years	↔ FN BMD, LS BMD, and TB BMD after adjustment
Total protein	Beasley et al., 2014 [148]	Prospective: Women’s Health Initiative clinical trials	144,580 postmenopausal women Dietary protein intake (% of E): Q1: <13.3; Q3: 14.2–14.8; Q5: ≥15.6.	6 years 50–79 years	↑ TB BMD and hip BMD with each 20% increase in protein intake ↔ LS BMD with protein intake
Total protein	Dawson-Hughes et al., 2004 [150]	Parallel RCT	32 healthy adults Protein intake (g/day): High protein: 57.6 ± 8.2; Low protein: 2.8 ± 0.5; All subjects: 800 mg of Ca.	9 weeks ≥50 years (high protein, 71.8 ± 9.8; low protein, 64.6 ± 10.8)	↑ TB BMC increased within high-protein group ↔ TB BMC between groups
Animal protein	Hunt et al., 1995 [151]	Parallel RCT	14 women Meat consumption (% of E): High meat (HM): 289 g (20%); Low meat (LM): 38.5 g (10%); Low meat with mineral supplement (LS).	7 weeks 51–70 years (62.9 ± 6.1)	↔ LS BMC and LS BMD
Soy protein vs. animal protein	Alekel et al., 2000 [159]	Parallel RCT: double blind	69 healthy perimenopausal women Intervention (g/day): Isoflavone soy protein (SPI) groups: 40 (soy protein) Control: 40 (whey protein) Co-intervention (/day): Isoflavone-rich soy protein (SPI+): 80.4 mg of aglycone components Isoflavone-poor soy protein (SPI−): 4.4 mg of aglycone components All subjects: 650 mg Ca	6 months 50.6 years	↑ LS BMD (5.6%) and LS BMC (10.1%) in the SPI+ group (treatment effect) ↑ LS BMD difference after adjustment for all covariates (SPI+ vs. whey; SPI+ vs. SPI plus whey; and SPI+ plus SPI vs. whey) ↑ LS BMC difference after adjustment for all covariates ((SPI+ vs. whey; SPI+ vs. SPI plus whey; and SPI+ plus SPI vs. whey)
Soy protein	Potter et al., 1998 [160]	Parallel RCT: double blind	66 postmenopausal women with hypercholesterolemia Intervention (g/day): Isolated soy protein with higher isoflavones (ISP 90): 40 of soy protein + high isoflavone (2.25 mg) Isolated soy protein with moderate isoflavones (ISP 52): 40 of soy protein + moderate isoflavone (1.39 mg) Control: casein and nonfat dry milk protein (CNFDM)	6 months intervention + 2 weeks basal lead-in period ISP 56: 49–73 years; ISP 90: 39–83 years; CNFDM: 51–74 years	↑ LS BMD, BMC after 6 months only in the ISP 90 group (vs. control) ↔ FN BMD, BMC; TB BMD, and BMC
Total protein	Thorpe et al., 2008 [161]	Parallel RCT	130 healthy, overweight adults Intervention (/day): Protein diet (P): 1.4 g/kg + three servings of dairy Carbohydrate diet (C): 0.8 g/kg + two servings of dairy	12 months 45.6 ± 8.9 years	↑ TB BMD in the P group (diet × time vs. the C group) ↑ TB BMD, LS BMD, and TH BMD in the P group (diet vs. C group) ↑ TB BMC in the P group (diet × time vs. the C group) ↑ LS BMC, TH BMC in the P group (diet vs. the C group)
Total protein	Dawson-Hughes et al., 2002 [162]	Parallel RCT	342 healthy older adults Intervention (/day): Treatment: 500 mg of Ca + 700 IU vitamin D Placebo: placebo Protein intake (% of total E) Q1: 9.64–15.49; Q2: 15.53–18.15; Q3: 18.16–29.14	3 years ≥65 years	↓ TB BMD, FN BMD loss with higher protein intake in the treatment group ↔ TB BMD loss with higher protein intake in the placebo group ↔ LS BMD
Total protein and animal protein	Hannan et al., 2000 [163]	Prospective: Framingham Osteoporosis Study	615 old adults Protein intake (g/day): Q1: 17–51; Q2: 52–67; Q3: 68–83; Q4: 84–152	4 years 68–91 years (75 ± 4.4)	↑ FN BMD, Ward BMD, and LS BMD loss in Q1 of total protein intake after multivariate adjustment (vs. Q4) ↔ troch BMD and radial shaft BMD loss in Q1 of total protein after multivariate adjustment (vs. Q4) ↑ FN BMD loss in Q1 and Q2 of animal protein intake after multivariate adjustment (vs. Q4) ↑ Ward BMD and LS BMD loss in Q1 of animal protein intake after multivariate adjustment (vs. Q4) ↔ troch BMD and radial shaft BMD loss in Q1 of animal protein intake after multivariate adjustment (vs. Q4)
Total protein and soy protein	Ho et al., 2008 [164]	Prospective: Framingham Osteoporosis Study	483 women Total protein (g/day): Q1: 12.5–34.5; Q2: 34.6–43.8; Q3: 43.9–56.1; Q4: 56.2–181.1. Soy protein (g/day): Q1: 0–1.06; Q2: 1.07–2.84; Q3: 2.85–5.71; Q4: 5.72–38.55	2.5 years 45–55 years (49.9 ± 2.7)	↔ LS BMD, FN BMD, TH BMC, and TB BMC with total protein and soy protein intake after adjustment for age–menopause stage and dietary E intake
Total protein	Promislow et al., 2002 [165]	Prospective: Rancho Bernardo Heart and Chronic Disease Study	960 adults	4 years 55–92 years (men: 70.0 ± 8.5; women: 71.2 ± 8.7)	↔ TH BMD, FN BMD, and LS BMD with total protein
Total protein	Recker et al., 1992 [166]	Prospective	156 healthy, nulliparous, young adult women	3.4 years 18.5–26 years (21.4 ± 1.7)	↔ LS BMD change rate with protein intake
Total protein	Sahni et al., 2014 [167]	Prospective: Framingham Offspring Study	1175 men and women Exposure: protein intake (% of E)	4.6 years 29–86 years (61 ± 9)	↔ FN BMD, LS BMD with protein after multivariate adjustment
Total protein	Li et al., 2010 [168]	Parallel RCT	70 healthy, overweight/obese adults Intervention (/day): High-protein-enriched (HP): 2.2 g/kg of LBM (30% of E) Standard protein (SP): 1.1 g/kg of LBM (15% of E)	13 months 49.4 ± 11.0 years	↔ TB BMD
Total protein	Gregg et al., 1999 [169]	Cross- sectional: Women’s Healthy Lifestyle Project (WHLP)	393 women	N/A 45–53 years (48.8 ± 1.8)	↑ BUA, SOS, and LS BMD with higher dietary protein intake ↔ FN BMD with higher dietary protein intake
Total protein	Lacey et al., 1991 [170]	Cross- sectional	178 Japanese women	N/A Premenopausal: 35–40 years (37.6 ± 2.01), postmenopausal: 55–60 years (58.0 ± 1.84)	↑ midradial BMC (r = 0.22; coefficient = 7.01) with % protein after adjusting for age, BMI, and kcal (for nutrients) among premenopausal women ↑ Correlation with protein and midradial BMC (r = 0.21; coefficient = 1.78) adjusting for age, BMI, and kcal (for nutrients) among postmenopausal women
Total protein	Metz et al., 1993 [171]	Cross- sectional	38 Caucasian women	N/A 24–28 years (25.9 ± 1.4)	↓ mid BMC (semipartial R^2^ = 0.153, regression coefficient = −0.503), distal BMC (semipartial R^2^ = 0.123, regression coefficient = −0.450) and distal BMD (semipartial R^2^ = 0.114, regression coefficient = −0.434) with protein intake ↔ mid BMD (semipartial R^2^ = 0.038, regression coefficient = −0.251) with protein intake
Total protein	Tylavsky et al., 1988 [172]	Cross- sectional	366 postmenopausal women Lacto-ovo-vegetarian (L) Omnivore (O)	N/A 60–98 years (L, 73.0 ± 0.8; O, 78.8 ± 0.4)	↑ distal BMC (β = 2.72) and mid BMC (β = 2.96) with protein intake ↔ distal BMD (β = 0.63) and mid BMD (β = 1.36) with protein intake
Total protein, dairy protein, nondairy protein, and vegetable protein	Langsetmo et al., 2017 [173]	Prospective: Osteoporotic in Men (MrOS)	5875 men Protein intake (% of E): Q1: 6.0–14.1; Q2: 14.2–15.8; Q3: 15.9–17.7; Q4: 17.8–29.3	10.5–11.2 years >65 years (73.6 ± 5.9)	↑ TH BMD with higher dairy protein (β = 0.10) and nondairy animal protein (β = 0.06) ↔ TH BMD with higher plant protein intake (β = −0.01)
MBP	Evans et al., 2007 [174]	Parallel RCT: double blind	43 healthy postmenopausal women Intervention (g/day): Soy protein isolate (SPI), SPI + exercise (SPI+Ex): 25.6 g of soy protein + 91.2 mg of isoflavone Milk protein isolate (MPI), MPI + exercise (MPI+Ex): 25.6 MPI All subjects: 900 mg of Ca, 125 IU vitamin D	9 months 62 ± 5 years	↔ BMD at any site in all groups after adjustment for covariates
Soy protein	Gallagher et al., 2004 [175]	Parallel RCT: double blind	50 postmenopausal women Intervention (g/day): SPI 96: 40 of soy protein + 96 mg of isoflavone; SPI 52: 40 of soy protein + 52 mg of isoflavone; SPI 4: 40 of soy protein + isoflavone (<4 mg)	15 months (intervention, 9 months; follow-up, 6 months) 40–62 years (55)	↔ LS BMD, FN BMD in all groups after adjusting for baseline u-NTX ↑troch BMD in SPI 4 at month 9 and 15 after adjusting for baseline u-NTX (vs. SPI 96; vs. SPI 52)
Soy protein	Lydeking-Olsen et al., 2004 [176]	Parallel RCT: double blind	89 postmenopausal Caucasian women Intervention (/day): Soy+: 17.5 g of soy protein + 76 mg of isoflavone Transdermal progesterone (TPD+): 25.7 mg of TPD Combined: Soy+, TPD+Placebo All subjects: food supplement	2 years 58.2 years	↓ LS BMD and LS BMC within the combined group and placebo group ↔ LS BMD and BMC within the Soy+, TDP+ group ↓ LS BMD and BMC in placebo (vs. Soy+) ↓ LS BMC in placebo (vs. TPD+) ↔ FN BMD or BMC
Total protein	Devine et al., 2005 [177]	Cross- sectional and longitudinal	1077 women not receiving pharmaceuticals that act on bone Protein intake (g/day): Low protein (T1): <66; Moderate protein (T2): 66–87; High protein (T3): >87	1 year >70 years (75 ± 3)	↑ BUA, BMD of all hip sites (TH, FN, troch, and intertrochanter) in T3 of protein intake after adjustment for age and BMI (vs. T1)
Total protein and animal protein	Pedone et al., 2010 [178]	Prospective: Invecchiare in Chianti (InCHIANTI) study	497 women	6 years 60–96 years (74.8 ± 7.5)	↑ total protein or animal protein/kg ideal weight with cortical BMD ↔ TB BMD and total trabecular BMD
Total protein and animal protein	Tucker et al., 2001 [179]	Prospective: Framingham Osteoporosis Study	855 adults Total protein intake (g/kg per d): Q1: not shown; Q4: 1.2–2.8 g/kg. Animal protein intake (g/kg per d): Q1: not shown; Q4: not shown	4 years 69–97 years	↑ FN BMD loss in Q1 and Q2 of protein intake after adjustment for sex and total caloric intake (vs. Q4) ↑ LS BMD loss in Q1 of protein intake after adjustment for sex and total caloric intake (vs. Q4) ↔ radial shift BMD loss in Q1 of protein intake after adjustment for sex and total caloric intake (vs. Q4) ↑ FN BMD loss in Q1 and Q2 of animal protein intake after multivariate adjustment (vs. Q4) ↑ LS BMD loss in Q1 of animal protein intake after multivariate adjustment (vs. Q4) ↔ radial shift BMD loss in Q1 of animal protein intake after multivariate adjustment (vs. Q4)
Total protein	Ballard et al., 2006 [180]	Parallel RCT	42 healthy adults Intervention (twice a day): Protein: 42 g of protein supplement; Control: isocaloric carbohydrate supplement	6 months 18–25 years	↔ total vBMD, trabecular vBMD, and TB BMC in the protein group after controlling for initial height, weight, and baseline bone values (vs. control)
Total protein	Meng et al., 2009 [181]	Prospective	862 community-dwelling women Protein intake (g/day): High protein (T3): >87; Moderate protein (T2): 66–87; Low protein (T1): <66.	5 years 70–85 years (75 ± 3)	↑ TB BMC (r = 0.15) with protein intake ↑ TB BMC in T3 after multivariate adjustment (vs. T1)
Total protein	Ho-pham et al., 2012 [182]	Prospective	181 women Total protein intake (mg/day): Vegans: 36; Omnivores: 62	2 years 61 ± 9.2 years	↔ LS BMD, FN BMD, and TB BMD rate of change between groups

BMC, bone mineral content; BMD, bone mineral density; BMI, body mass index; BUA, broadband ultrasound attenuation; Ca, calcium; E, energy; FFQ, food frequency questionnaire; FN, femoral neck; g, gram; LBM, lean body mass; LS, lumbar spine; MBP, milk basic protein; N, number; N/A, not available; RCT, randomized controlled trial; SE, standard error; SOS, speed of sound; TB, total body; TH, total hip; troch, trochanter; u-NTX, urinary N-telopeptide of type 1 collagen; vBMD, volumetric bone mineral density; Ward, Ward’s triangle; ↑, increase; ↓, decrease; ↔, no effect.

**Table 4:** The effects of proteins on bone fracture outcomes in individual human studies.

Nutrient Type	Ref	Study Type	N of Subjects Study Design	Follow-Up Period and Age	Bone Fracture Outcomes
Total protein	Munger et al., 1999 [91]	Prospective study: Iowa Women’s Health Study	32,050 postmenopausal women Total protein (g/MJ): Q1: <9.56; Q2: 9.56–10.78; Q3: 10.78–12.05; Q4: >12.05	3 years 55–69 years	↔ hip fracture risk in Q4 after multivariate adjustment (vs. Q1)
Animal protein			32,050 postmenopausal women Animal protein (g/MJ) Q1: <6.48; Q2: 6.48–7.82; Q3: 7.82–9.26; Q4: >9.26	3 years 55–69 years	↓ hip fracture risk by 69% in Q4 after multivariate adjustment (vs. Q1)
Vegetable protein			32,050 postmenopausal women Vegetable protein (g/MJ) Q1: <2.51; Q2: 2.51–2.88; Q3: 2.88–3.28; Q4: >3.28	3 years 55–69 years	↔ hip fracture risk in Q4 after multivariate risk adjustment (vs. Q1)
Total protein	Langsetmo et al., 2015 [119]	Prospective: Canadian Multicentre Osteoporosis Study	4661 adults Protein intake (% of E): Q1: <12.6; Q2: 12.6–14.1; Q3: 14.1–15.7; Q4: >15.7	13 years >50 years	↔ main fracture risk in Q4 of protein intake after multivariate risk adjustment among men and women (vs. Q1)
Total protein	Misra et al., 2011 [120]	Prospective: Framingham Osteoporosis Study	946 adults Protein intake (g/day): Q1: 46.45; Q2: 59.61; Q3: 67.70; Q4: 82.74	17 years 28–62 years	↔ hip fracture risk in Q4 of protein intake (vs. Q1)
Total protein, animal protein and vegetable protein	Sahni et al., 2010 [121]	Prospective: Framingham Offspring Study	3656 adults Protein intake (g/day): <800 mg of Ca intake Total protein: Data not shown Animal protein: T1, 34; T3, 60 Vegetable protein: Data not shown ≥800 mg of Ca intake Total protein: T1, 79; T3, 103 Animal protein: T1, 48; T3, 76 Vegetable protein: T1, 22; T3, 34	12 years 55 years (men: 55.3 ± 9.9; women: 54.9 ± 9.8)	↔ hip fracture risk in T3 of total protein and vegetable protein intake after multivariate risk adjustment with total Ca intake <800 mg/day (vs. T1) ↑ hip fracture risk by 217% in T3 of animal protein intake after multivariate risk adjustment with total Ca intake <800 mg/day (vs. T1) ↔ hip fracture risk in T3 of total protein, animal protein, and vegetable protein intake after multivariate risk adjustment with total Ca intake ≥800 mg/day (vs. T1)
Total protein, animal protein, vegetable protein, and animal protein/vegetable protein ratio	Martinez et al., 2012 [122]	Case– control	334 patients who suffered a low-energy fracture 6–24 months before the inclusion and controls Total protein (g/day): Q1: <85; Q2: 85–99; Q3: 100–117; Q4: >118. Animal protein (g/day): Q1: <48; Q2: 49–63; Q3: 64–73; Q4: 74–87 Vegetable protein (g/day): Q1: <30; Q2: 31–34; Q3: 35–39; Q4: 40–47	N/A ≥65 years (cases: 73.2, controls: 71.2)	↓ low-energy fracture by 62% in T3 of animal/vegetable protein ratio after multivariate adjustment (vs. T1) ↔ low-energy fracture in Q4 of total, animal, and vegetable protein intake after multivariate adjustment (vs. Q1)
Total protein	Nieves et al., 1992 [123]	Case– control	329 white women with first hip fracture and controls Protein intake (g/day): Q1: 0–24; Q2: 25–34; Q3: 35–44; Q4: 45–54; Q5: ≥55	N/A 50–103 years	↔ hip fracture
Total protein, animal protein and vegetable protein	Wengreen et al., 2004 [124]	Case–control	2501 adults (cases with hip fracture or controls) Total protein intake (% of E): Q1: 5.6–13.9; Q2: 14.0–15.5; Q3: 15.6–17.3; Q4: 17.4–30.8 Animal protein intake (% of E): Q1: 0.0–8.2; Q2: 8.3–9.9; Q3: 10.0–11.7; Q4: 11.8–23.6. Vegetable protein intake (% of E): Q1: 0.0–5.0; Q2: 5.1–5.6; Q3: 5.7–6.2; Q4: 6.3–14.7	N/A 50–89 years	↓ hip fracture by 65% in Q4 of total protein intake among subjects aged 50–69 years after multivariate adjustment (P_trend_ < 0.001) ↓ hip fracture by 57% in Q4 of animal protein intake among subjects aged 50–69 years after multivariate adjustment (P_trend_ = 0.21) ↓ hip fracture by 48% in Q4 of vegetable protein intake among subjects aged 50–69 years after multivariate adjustment (P_trend_ = 0.19) ↔ hip fracture with any type of protein intake among subjects aged 70–89 years
MBP	Meyer et al., 1997 [128]	Prospective	39,787 middle-aged adults Milk consumption (glasses/day): ≤1 vs. ≥4 Nondairy animal protein (men/women) (g/day): Q1: <14.2/<13.6; Q2: 14.2–17.6/13.6–16.9; Q3: 17.6–21.6/16.9–20.6; Q4: >21.6/>20.6	11.4 years 35–49 years (men, 47.1 ± 4.5; women, 47.1 ± 4.6)	↔ hip fracture risk in ≥4 among women after multivariate adjustment (vs. ≤1) ↓ hip fracture risk by 54% in ≥4 among men after multivariate adjustment (vs. ≤1) ↔ hip fracture risk in Q4 of nondairy animal protein intake among women and men after multivariate adjustment (vs. Q1)
Total protein	Feskanich et al., 1996 [129]	Prospective: Nurses’ Health Study (NHS)	85,900 Caucasian females aged 34–59 years Total protein intake (g/day): Q1: <68; Q2: 68–77; Q3: 78–85; Q4: 86–95; Q5: >95	12 years 30–65 years	↔ hip fracture in Q5 of total protein intake in multivariate model (vs. Q1) ↑ forearm fracture by 22% in Q5 of total protein intake in multivariate model (vs. Q1)
Animal protein			85,900 Caucasian females aged 34–59 years Animal protein intake (g/day): Q1: <51; Q2: 52–61; Q3: 62–69; Q4: 70–80; Q5: >80		↔ hip fracture in Q5 of animal protein intake in multivariate model (vs. Q1) ↑ forearm fracture by 25% in Q5 of animal protein intake in multivariate model (vs. Q1)
			Women aged 40–65 years Animal protein intake during teenage years (g/day): Q1: ≤30; Q2: 31–45; Q3: 46–55; Q4: 56–70; Q5: >70 Beef, pork, or lamb intake during teenage years (servings/week): Q1: ≤1; Q2: 2–4; Q3: 5–6; Q4: ≥7.		↔ hip fracture and forearm fracture with highest daily intake of animal protein (vs. lowest) ↔ hip fracture and forearm fracture with highest serving of animal foods (vs. lowest)
Vegetable protein			85,900 Caucasian females aged 34–59 years Vegetable protein intake (g/day): Q1: <12; Q2: 12–14; Q3: 15–16; Q4: 17–19; Q5: >19.		↔ hip fracture and forearm fracture risk in Q5 of vegetable protein intake in multivariate model (vs. Q1)
Total protein	Dargent-Molina et al., 2008 [130]	Prospective: E3N (Etude Epidémiologique de femmes de la Mutuelle Générale de l’Education Nationale (MGEN))	36,217 postmenopausal women Total protein intake (g/1000 kcal/day): Q1: <40.75; Q2: 40.75–45.16; Q3: 45.16–50.11; Q4: >50.11	12 years (8.37 ± 1.73) 40–65 years	↔ fracture risk with total protein intake in overall population after multivariate adjustment ↑ fracture risk by 51% in Q4 of total protein intake in lowest Ca quartile after multivariate adjustment (vs. Q1)
Animal protein			36,217 postmenopausal women Animal protein intake (g/1000 kcal/day): Q1: <22.42; Q2: 22.42–27.75; Q3: 27.75–33.52; Q4: >33.52.		↔ fracture risk with animal protein intake in overall population after multivariate adjustment ↑ fracture risk by 66% in Q4 of animal protein intake in low-Ca quartile after multivariate adjustment (vs. Q1)
Vegetable protein			36,217 postmenopausal women Vegetable protein intake (g/1000 kcal/day): Q1: <10.07; Q2: 10.07–12.01; Q3: 12.01–14.12; Q4: >14.12.		↔ fracture risk with vegetable protein intake in overall population after multivariate adjustment ↓ fracture risk by 32% in Q4 of vegetable protein intake in low-Ca quartile after multivariate adjustment (vs. Q1)
Total protein by weight			36,217 postmenopausal women Total protein intake by weight (g/kg/day): Q1: <1.15; Q2: 1.15–1.41; Q3: 1.41–1.71; Q4: >1.71.		↔ fracture risk in Q4 of total protein by weight in overall population after multivariate adjustment (vs. Q1) ↑ fracture risk 46% in Q4 of total protein by weight in lowest quartile for Ca intake (vs. Q1)
Total protein	Mussolino et al., 1998 [131]	Prospective: NHANES Epidemiologic Follow-Up Study	2249 Caucasian men Protein intake (g/day): Q1: <56; Q2: 56–73; Q3: 74–97; Q4: >97	13.9 years 45–74 years	↔ hip fracture risk in Q4 of protein intake after multivariate risk adjustment (vs. Q1)
Total protein	Beasley et al., 2014 [148]	Prospective: Women’s Health Initiative clinical trials	144,580 postmenopausal women Dietary protein intake (% of E): Q1: <13.3; Q3: 14.2–14.8; Q5: ≥15.6	6 years 50–79 years	↔ hip fracture, LS fracture, and total fracture in higher than 20% protein intake per E ↓ forearm fracture by 7% in higher than 20% protein intake per E
Total protein	Fung et al., 2017 [149]	Prospective: Nurses’ Health Study (NHS)	109,882 postmenopausal women and men Total protein intake (men/women) (g/day): Q1: 73.6/60.2; Q2: 83.1/68.0; Q3: 89.9/73.5; Q4: 97.1/79.3; Q5: 108.3/88.6	22 years Men: ≥50 years Women: menopause	↓ hip fracture in Q5 of total protein intake among men after multivariable adjustment (RR for each 10 g increase = 0.92) (vs. Q1) ↔ hip fracture in Q5 of total protein intake among women after multivariable adjustment (vs. Q1) ↔ hip fracture risk in Q5 of total protein in pooled men and women (vs. Q1)
Animal protein			Animal protein intake (men/women) (g/day): Q1: 46.2/39.0; Q2: 56.3/47.0; Q3: 63.5/52.8; Q4: 71.3/59.0; Q5: 83.6/60.7		↓ hip fracture by 9% with Q5 of animal protein intake among men after multivariable adjustment (vs. Q1) ↔ hip fracture risk in Q5 of animal protein among women after adjustment for multivariable (vs. Q1) ↓ hip fracture risk by 5% in Q5 of animal protein in pooled men and women (vs. Q1)
Vegetable protein			Plant protein intake (men/women) (g/day) Q1: 19.6/14.7; Q2: 23.2/17.9; Q3: 25.8/19.9; Q4: 28.6/21.8; Q5: 33.4/25.1		↔ hip fracture in Q5 of plant protein intake among men after multivariable adjustment (vs. Q1) ↔ hip fracture in Q5 of plant protein intake among women after multivariable adjustment (vs. Q1) ↓ hip fracture risk in Q5 of plant protein intake (RR for each 10 g increase = 0.88) in pooled men and women (vs. Q1)
MBP			Dairy protein intake (g/day) Men: Q1: 6.8; Q2: 10.6; Q3: 14.0; Q4: 18.2; Q5: 26.5. Women: Q1: 6.8; Q2: 10.6; Q3: 13.8; Q4: 17.8; Q5: 24.6		↔ hip fracture in Q5 of dairy protein intake among men after multivariable adjustment (vs. Q1) ↔ hip fracture in Q5 of dairy protein intake among women after multivariable adjustment (vs. Q1) ↓ hip fracture risk in Q5 of dairy protein intake (RR for each 10 g increase = 0.91) in pooled men and women (vs. Q1)
Total protein, dairy protein, nondairy protein, and vegetable protein	Langsetmo et al., 2017 [173]	Prospective: Osteoporotic in Men (MrOS)	5875 men Protein intake (% of E): Q1: 6.0–14.1; Q2: 14.2–15.8; Q3: 15.9–17.7; Q4: 17.8–29.3	10.5–11.2 years >65 years (73.6 ± 5.9)	↓ low-trauma fracture by 8%, hip fracture by 16% with Q4 of total protein intake after multivariate adjustment (vs. Q1) ↓ low-trauma fracture by 7%, hip fracture by 20% with Q4 of dairy protein intake after multivariate adjustment (vs. Q1) ↓ hip fracture by 16% with Q4 of nondairy protein after multivariate adjustment (vs. Q1) ↔ all types of fracture with Q4 of plant protein after multivariate adjustment (vs. Q1)
Total protein	Ho-pham et al., 2012 [182]	Prospective	181 women Total protein intake (mg/day): Vegans: 36; Omnivores: 62	2 years 61 ± 9.2 years	↔ fracture incidence in groups
Soy protein	Koh et al., 2009 [183]	Prospective: Singapore Chinese Health Study	63,154 adults Soy protein intake (g/day): Q1: <2.7; Q2: 2.7–4.7; Q3: 4.7–7.6; Q4: >7.6	8 years 45–74 years	↔ hip fracture risk in Q4 of soy protein intake among men (vs. Q1) ↓ hip fracture risk by 21% in Q4 of soy protein intake among women (vs. Q1)
Soy protein	Zhang et al., 2005 [184]	Prospective study Study of Osteoporotic Fracture	24,403 postmenopausal women Soy protein intake (g/day): Q1: <4.98; Q2: 4.98–7.32; Q3: 7.33–9.77; Q4: 9.78–13.26; Q5: ≥13.27	5 years 40–70 years (60)	↓ hip fracture risk by 37% in Q5 of protein intake after multivariate risk adjustment (vs. Q1)
Total protein	Cauley et al., 2016 [185]	Prospective: Osteoporotic Fractures in Men Study (MrOS)	5876 men Exposure: protein intake (% of E)	8.6 years >65 years	↓ hip fracture risk by 19% with protein intake

Ca, calcium; E, energy; g, gram; LS, lumbar spine; MBP, milk basic protein; N, number; N/A, not available; ↑, increase; ↓, decrease; ↔, no effect.

**Table 5:** The effects of proteins on bone turnover marker outcomes in individual human studies.

Nutrient Type	Ref	Study Type	N of Subjects Study Design	Follow-Up Period and Age	BTM Outcomes
Total protein	Cao et al., 2011 [73]	Crossover RCT	16 postmenopausal women Protein intake (/day): High-protein, high-PRAL diet (HPHP diet): 118 g of protein and 33 mEq of PRAL Low-protein, low-PRAL diet (LPLP diet): 61 g, −48 mEq	7 weeks (each separated by 1 week break) 40–75 years (56.9 ± 3.2)	↑ serum IGF-1, Ca absorption, and urinary Ca excretion in HPHP diet (vs. LPLP diet) ↓ serum i-PTH decreased in HPHP diet (vs. LPLP diet) ↔ u-NTX, urinary DPD, serum biomarkers (Ca, TRAP, Cr, CTX, OC, OPG, and sRANKL) between the two diets
Total protein	Kerstetter et al., 1997 [82]	Parallel RCT	16 healthy premenopausal women Protein intake (g/kg): High protein intake: 2.1; Medium protein intake: 1.0 Low protein intake: 0.7	4 days 20–40 years (26.7 ± 1.3)	↑ serum ionized Ca in the low-protein diet (vs. medium) ↔ urinary fractional Ca excretion in the low-protein diet (vs. medium) ↑ midmolecule PTH, i-PTH, calcitriol, and NcAMP excretion in the low-protein diet (vs. moderate) ↓ urinary Ca excretion in the low-protein diet (vs. the medium-protein diet) ↑ urinary Ca and urinary fractional Ca excretion in the high-protein diet (vs. the medium-protein diet) ↔ midmolecule PTH, i-PTH, calcitriol, and NcAMP excretion in the high-protein diet (vs. moderate-protein diet) ↔ serum total Ca within all diets
Total protein	Rapuri et al., 2003 [109]	Cross- sectional and prospective	473 postmenopausal women Exposure: protein intake (% of E) Q1: 13.1 ± 0.12; Q2: 15.1 ± 0.11; Q3: 16.7 ± 0.12; Q4: 19.8 ± 0.12	N/A 65–77 years	Cross-sectional analysis: ↔ serum Ca, ionized Ca, P, ALP, albumin, i-PTH, 25(OH)D, 1,25(OH)_2_D, OC, urinary Ca:Cr, and u-NTX:Cr Prospective analysis: ↔ serum Ca, ALP, i-PTH, 25(OH)D, 1,25(OH)_2_D and OC, Ca absorption, and u-NTX:Cr
Total protein	Tkatch et al., 1992 [116]	Parallel RCT	48 elderly men and women Intervention (g/day): Protein: 20.4; control: 0	7 months ≥60 years (82)	↑ plasma OC within the protein group
MBP	Kerstetter et al., 2015 [117]	Parallel RCT: double blind	208 men and women Intervention (g/day): Whey protein: 45 of whey protein Control: 0 All subjects: 400 IU vitamin D	18 months men: ≥70 years women: ≥60 years	↔ serum P1NP, OC between the groups ↑ serum CTX in the whey protein group (vs. control) ↑ serum IGF-1 in the whey protein group (vs. control)
MBP	Zhu et al., 2011 [118]	Parallel RCT: double blind	186 healthy ambulant postmenopausal women Protein intake (g/day): Protein: 30 (whey protein + skim milk); Placebo: 2.1 (skim milk)	2 years 70–80 years (74.3 ± 2.7)	↑ serum IGF-1 at 1 year and 2 years in the protein group (vs. control)
MBP	Aoe et al., 2005 [125]	Parallel RCT: double blind	27 healthy menopausal women Protein intake (mg/day): MBP group: 40; placebo group: 0	6 months 50.5 ± 3.0 years	↓ u-NTX in the MBP group (vs. placebo) ↔ OC
MBP	Uenishi et al., 2007 [126]	Parallel RCT: double blind	35 healthy young women Protein intake (mg/day): MBP: 40; Placebo: 0	6 months 21.3 ± 1.2 years	↓ u-NTX in the MBP group (vs. placebo) ↑ serum OC in the MBP group (vs. placebo)
MBP	Zou et al., 2009 [127]	Parallel RCT	81 healthy young women Intervention (/day): MBP (40 mg of milk) group: 250 mL of whole milk + 40 mg of MBP Whole-milk group: 250 mL Whole-milk control group: N/A	8 months 19.6 ± 0.6 years	↓ serum NTX within the MBP group at 8 months and the whole-milk group at 6 months ↔ serum NTX between MBP and whole milk ↔ BALP within both the MBP and whole-milk groups
Total protein	Jesudason et al., 2013 [133]	Parallel RCT	136 postmenopausal women Protein intake (g/day) High protein (HP): >90 High normal protein (HNP): <80	24 months 40–70 years (HP: 59.5 ± 0.4; HNP: 59.4 ± 0.4)	↔ PTH, serum ALP in the HP group (vs. the HNP group) ↓ 25(OH)D in the HP group (time, diet vs. the HNP group) ↓ CTX in the HP group (time, diet, diet × time vs. the HNP group) ↓ OC in the HP group (time, diet × time vs. the HNP group) ↑ urine Ca in the HP group (time, diet × time vs. the HNP group)
MBP	Kukuljan et al., 2009 [134]	Parallel RCT	175 healthy men Protein intake (g/day): Milk: 13.2; Control: 0	12 months 50–79 years (MBP: 61.7 ± 7.7; control: 59.9 ± 7.4)	↑ serum 25(OH)D in the milk group (vs. control) ↔ PTH
Total protein	Sukumar et al., 2011 [135]	Parallel RCT	47 healthy overweight/obese postmenopausal women Protein intake (% of E): HP: 30; NP: 18	1 year 58 ± 4 years	↔ OC
MBP	Flodin et al., 2014 [137]	Parallel RCT	67 patients with a recent hip fracture Intervention (/day): Bisphosphonates + nutritional supplementation (BN): 40 g of MBP + 5 mg of risedronate Bisphosphonates (B): 0 g of MBP + 5 mg of risedronate Controls (C): placebo All subjects: 1000 mg of Ca + 800 IU vitamin D_3_	1 year >60 years (79 ± 9)	↔ CTX
MBP	Bharadwaj et al., 2009 [138]	Parallel RCT	31 healthy postmenopausal women Intervention (/day): Ribonuclease-enriched lactoferrin (R-ELF): 250 mg of R-ELF from milk; control: 0 All subjects: 100% RDA of Ca	6 months 45–60 years (R-ELF, 53.5 ± 5.4; Control, 51.0 ± 4.4)	↑ OC within the R-ELF group (vs. control)
MBP	Holm et al., 2008 [139]	Parallel RCT: double blind	29 healthy, early postmenopausal women Intervention (/day): Nutrient (NUT): 10 g of whey protein, 31 g of carbohydrate, 1 g of fat, 5.0 μg of vitamin D, and 250 mg of Ca Control (C): 6 g of carbohydrate and 12 mg of Ca	24 weeks Nut: 55 ± 1 years C: 55 ± 1 years	↑ serum OC in NUT at week 12 and 24 (vs. C) ↔ CTX
MBP	Schürch et al., 1998 [140]	Parallel RCT: double blind	82 orthopedic patients with recent hip fracture Intervention (g/day): Protein: 20 of milk protein (5 days/week); Control: 0	12 months >60 years (protein: 81.1 ± 7.4; control: 80.2 ± 7.4)	↑ IGF-1 changes in the protein group at month 6 (vs. control) ↔ OC, PTH, 1,25(OH)_2_D, PD:Cr, and DPD:Cr between the groups
MBP	Tengstrand et al., 2007 [141]	Parallel RCT	52 lean, postmenopausal patients with recent FN fracture Intervention (g/day): Nutrition (PR) and combined therapy (PR/N): 20 Controls (C): 0 All subjects: 1 g of Ca + 800 IE vitamin D	12 months 70–92 years (83 ± 5)	↑ OC within the PR group at month 6 and 12 ↔ CTX within the PR group
Soy protein	Arjmandi et al., 2005 [143]	Parallel RCT: double blind	62 postmenopausal women Intervention (/day): Soy: 25 g of soy protein + 60 mg of isoflavones Control: 25 g of non-soy protein	1 year <65 years (soy: 53 ± 6; control: 56 ± 5)	↑ IGF-I in the soy group (vs. control) ↔ OC, BSAP, ALP, and urinary DPD
Soy protein	Kenny et al., 2009 [144]	Parallel RCT: double blind	97 healthy ambulatory postmenopausal women Intervention (/day): Soy protein placebo (SPI−), soy protein isoflavones (SPI+): 18 g of soy protein Control protein placebo, control protein isoflavones: 18 g of milk and egg white protein Co-intervention (/day): SPI+: 35 mg isoflavones All subjects: if not achieving 1200–1500 mg of Ca via diet, they were administered 315 mg of Ca and 200 IU vitamin D	1 year >60 years (73.1 ± 5.9)	↔ BSAP, u-NTX/Cr between the groups
Soy protein	Kreijkamp et al., 2004 [145]	Parallel RCT: double blind	175 healthy postmenopausal women Intervention (g/day): Soy protein + isoflavones (SPI+): 25.6 of isoflavone-rich soy protein Placebo: 25.6 of milk protein	1 year 60–75 years (SPI+, 66.5 ± 4.7; placebo, 66.7 ± 4.8)	↔ BSAP in the SPI+ group (vs. placebo)
Soy protein and MBP	Vupadhyayula et al., 2009 [146]	Parallel RCT: double blind	157 healthy postmenopausal women Intervention (g/day): Soy protein: 25 of soy protein isolate; soy protein + isoflavone: 25 of soy protein isolate + 90 mg of isoflavones; milk protein: 25 of casein and whey	2 years Soy protein: 63.6 ± 0.6 years Soy protein + isoflavone: 63.4 ± 0.6 years Milk protein: 63.8 ± 0.5 years	↔ u-NTX
Total protein	Dawson-Hughes et al., 2004 [150]	Parallel RCT	32 healthy adults Protein intake (g/day): High protein: 57.6 ± 8.2; Low protein: 2.8 ± 0.5. All subjects: 800 mg of Ca	9 weeks ≥50 years (high protein, 71.8 ± 9.8; low protein, 64.6 ± 10.8)	↑ serum IGF-1 in high-protein group over the period of 35–49 days or 63 days ↓ u-NTX in high-protein group over the period of 35–49 days or 63 days ↔ serum OC, PTH
Animal protein	Hunt et al., 1995 [151]	Parallel RCT	14 women Meat consumption (% of E): High meat (HM): 289 g (20%); Low meat (LM): 38.5 g (10%). Low meat with mineral supplement (LS)	7 weeks 51–70 years (62.9 ± 6.1)	↔ Ca balance, urinary Ca, serum Ca, ionized Ca, and 25(OH)D ↓ serum ALP in the HM group (vs. LM)
Total protein	Jenkins et al., 2003 [152]	Crossover RCT	20 men and postmenopausal women Total protein (g/day) High protein (HP): 189 ± 12; Control: 111 ± 6	4.3 weeks 35–71 years (56 ± 8.5)	↔ serum Ca between groups ↔ PTH, BSAP, 25(OH)D, and u-NTX ↑ urinary Ca excretion in the HP group (vs. control)
Total protein	Kerstetter et al., 1998 [153]	Parallel RCT	12 premenopausal women Protein intake (g/kg): High protein intake: 2.1 (134.9 g/day); Low protein intake: 0.7 (45.8 g/day)	5 days 21–39 years (26.0 ± 1.8)	↔ total or ionized serum Ca between the two diets ↔ fractional urinary Ca excretion in the high-protein diet (vs. low) ↑ urinary Ca in the high-protein diet (vs. low) ↑ serum PTH, 1,25(OH)_2_D in the low-protein diet (vs. high) ↓ fractional intestinal Ca absorption and true Ca absorption in the low-protein (vs. high-protein) diet
Total protein	Kerstetter et al., 2000 [154]	Parallel RCT	Eight premenopausal women One of four amounts of protein (g/kg/day): 1. 0.7 (44.3 g/day); 2. 0.8 (50.2 g/day); 3. 0.9 (56.7 g/day); 4. 1.0 (62.7 g/day)	4 days 20–40 years (23.1 ± 2.3)	↔ serum Ca, urine Ca between four protein intakes ↓ NcAMP was lower with 0.8 g/kg of protein intake (vs. 0.7 g/kg intake) (*p* < 0.05) ↓ i-PTH, calcitriol, and NcAMP lower with 0.9 g/kg of protein intake (vs. 0.8 g/kg of protein) ↓ midmolecule PTH lower with 0.9 g/kg of protein intake (vs. 0.8 g/kg of protein) (*p* < 0.0001)
Total protein, animal protein and soy protein	Kerstetter et al., 2006 [155]	Parallel RCT	20 pre- and postmenopausal women Protein levels (g/kg): high protein, 2.1; low protein, 0.7 Protein types: meat and soy Median protein intake (g/day): Meat: high: 102.7 ± 3.4; low: 20.7 ± 1.1 Soy: high: 88.8 ± 2.9; low: 21.8 ± 0.8	4 days 20–66 years (29.2 ± 1.8)	↑ urinary Ca and fractional Ca excretion in high-protein diets (vs. low-protein diets) ↔ urinary Ca or fractional Ca excretion (levels × types of protein) ↑ serum PTH in low-protein (vs. high-protein) and soy diets (vs. meat diets) ↔ PTH between protein level and protein type ↑ NcAMP in the soy diet (vs. meat) and with higher soy protein intake (vs. low soy) ↑ serum calcitriol concentration in the soy diet (vs. meat) ↔ u-NTX in the levels of protein and types of diet ↔ Ca absorption in the soy diet (vs. meat diet)
Total protein	Pannemans et al., 1997 [156]	Crossover RCT	55 young and elderly adults Protein intake (% of total energy): Low-protein diet (Diet A): 12; High-protein diet (Diet B): 21.	3 weeks Young adults: 29.3 years; elderly adults: 70.1 years	↓ urinary Ca excretion in Diet A among young subjects and all subjects (vs. Diet B)
Total protein	Kerstetter et al., 1999 [157]	Parallel RCT	16 healthy premenopausal women Protein intake (g/kg): High protein intake: 2.1; Moderate protein intake: 1.0; Low protein intake: 0.7	4 days 20–40 years (26.7 ± 1.3)	↑ serum midmolecule PTH, i-PTH, 1,25(OH)_2_D, and NcAMP in low-protein diet (vs. moderate) ↔ calcitropic hormone within the moderate-protein diet ↔ i-PTH, 1,25(OH)_2_D, and NcAMP within the high-protein diet ↑ u-NTX excretion in the high-protein diet (vs. low) ↔ OC in all groups ↑ BSAP in the low-protein group (vs. moderate) ↔ BSAP in the high protein (vs. low; vs. moderate)
Soy protein vs. animal protein	Alekel et al., 2000 [159]	Parallel RCT: double blind	69 healthy perimenopausal women Intervention (g/day): Isoflavone soy protein (SPI) groups: 40 of soy protein Control: 40 of whey protein Co-intervention (/day): Isoflavone-rich soy protein (SPI+): 80.4 mg of aglycone components Isoflavone-poor soy protein (SPI−): 4.4 mg of aglycone components All subjects: 650 mg of Ca	6 months 50.6 years	↔ BSAP, NTX
Total protein	Li et al., 2010 [168]	Parallel RCT	70 healthy, overweight/obese adults Intervention (/day): High-protein enriched (HP): 2.2 g/kg of LBM (30% of E) Standard protein (SP): 1.1 g/kg of LBM (15% of E)	13 months 49.4 ± 11.0 years	↔ urine Ca, serum Cr
MBP	Evans et al., 2007 [174]	Parallel RCT: double blind	43 healthy postmenopausal women Intervention (g/day): Soy protein isolate (SPI), SPI + exercise (SPI+Ex): 25.6 of soy protein + 91.2 mg of isoflavone Milk protein isolate (MPI), MPI + exercise (MPI+Ex): 25.6 of MPI All subjects: 900 mg of Ca, 125 IU vitamin D	9 months 62 ± 5 years	↓ serum BSAP, CTX in the SPI groups after adjustment for covariates (vs. MPI)
Soy protein	Gallagher et al., 2004 [175]	Parallel RCT: double blind	50 postmenopausal women Intervention (g/day): SPI 96: 40 of soy protein + 96 mg of isoflavones; SPI 52: 40 of soy protein + 52 mg of isoflavones; SPI 4: 40 of soy protein + isoflavones (<4 mg)	15 months (intervention, 9 months; follow-up, 6 months) 40–62 years (55)	↔ serum OC, u-NTX within the groups
Soy protein	Lydeking-Olsen et al., 2004 [176]	Parallel RCT: double blind	89 postmenopausal Caucasian women Intervention (/day): Soy+: 17.5 g of soy protein + 76 mg of isoflavones Transdermal progesterone (TPD+): 25.7 mg TPD Combined: Soy+, TPD+Placebo All subjects: food supplement	2 years 58.2 years	↔ P1NP, ICTP, or the P1NP/ICTP ratio
Total protein	Ho-pham et al., 2012 [182]	Prospective	181 women Total protein intake (mg/day): Vegans: 36; omnivores: 62	2 years 61 ± 9.2 years	↔ CTX, P1NP between the groups
MBP	Aoe et al., 2001 [186]	Parallel RCT	33 healthy adult women Intervention (mg/day): MBP: 40 MBP; placebo: 0	6 months 28.8 ± 8.7 years	↓ u-NTX, P1NP/Cr, DPD/Cr in MBP group (vs. placebo) ↔ serum OC, BSAP
Soy protein	George et al., 2020 [187]	Parallel RCT: double blind	88 healthy adults Intervention(g/day): Soy: 40 of soy protein + 96 mg of isoflavones; control: 40 of casein	3 months 27–87 years (soy, 60.3 ± 12.0; control, 60.6 ± 12.0)	↑ IGF-1 within and between the groups ↔ serum estradiol, TRAP ↓ BSAP within the soy group
Total protein	Ince et al., 2004 [188]	Parallel RCT	39 healthy premenopausal women consuming ad libitum diets Intervention (/day): Recommended dietary allowance (RDA): isocaloric diet containing US RDA protein (0.8 g/kg); ad libitum: free diet	2 weeks (1 week ad libitum, 1 week RDA) 22–39 years (27.3 ± 1.8)	↓ urinary Ca, u-NTX after RDA treatment ↔ serum Ca, OC, PTH, and 1,25(OH)_2_D
Soy protein	Murray et al., 2003 [189]	Parallel RCT: double blind	30 healthy postmenopausal women Intervention(/day): Group 1: 0.5 mg of estradiol + placebo; Group 2: 1.0 mg of estradiol + placebo; Group 3: 0.5 mg of estradiol + 25 g of SPI with 120 mg of isoflavones; Group 4: 1.0 mg of estradiol + 25 g of SPI with 120 mg of isoflavones	6 months >45 years (Group 1, 53.0 ± 3.4; Group 2, 53.4 ± 4.1; Group 3, 56.3 ± 7.4; Group 4, 56.6 ± 9.1)	↔ serum NTX

1,25(OH)2D, 1,25-dihydroxyvitamin D; 25(OH)D, 25-hydroxyvitamin D; ALP, alkaline phosphatase; BALP, bone alkaline phosphatase; BSAP, bone-specific alkaline phosphatase; BTM, bone turnover marker; Ca, calcium; Cr, creatinine; CTX, C-terminal telopeptide cross-link of type 1 collagen; DPD, deoxypyridinoline; E, energy; g, gram; ICTP, type 1 C-terminal telopeptide; IGF-1, insulin-like growth factor-1; i-PTH, intact parathyroid hormone; LBM, lean body mass; MBP, milk basic protein; N, number; N/A, not available; NcAMP, nephrogenous cyclic adenosine monophosphate; NTX, N-telopeptide of type 1 collagen; OC, osteocalcin; OPG, osteoprotegerin; P, phosphorus; P1NP, type 1 procollagen-N-propeptide; PD, pyridinoline; PRAL, potential renal acid load; PTH, parathyroid hormone; RCT, randomized controlled trial; sRANKL, soluble receptor activator nuclear factor-kB ligand; TRAP, tartrate-resistant acid phosphatase; u-NTX, urinary N-telopeptide of type 1 collagen; ↑, increase; ↓, decrease; ↔, no effect.

**Table 6:** The effects of fats on bone outcomes in meta-analysis of human studies.

Ref	Nutrient Type	Description	Studies	Study Type; N of Subjects	Follow-Up Period and Age Range or Mean Age	BMD and/or Bone Fracture and/or BTM Outcomes
Dou et al., 2022 [196]	N-3 PUFA	A meta- analysis of BMD outcomes	Six studies [197,198,199,200,201,202]	RCT; 491 subjects	3 to 36 months 25–85 years	↑ BMD with N-3 PUFA (WMD = 0.01; 95% CI 0.00 to 0.01 g/cm^2^; *I*^2^ = 27.4%; P_het_ = 0.219)
		Four meta- analyses of BTM outcomes	Seven studies [197,200,203,204,205,206,207]	RCT; 475 subjects	6 weeks to 18 months 25–85 years	↔ BSAP with N-3 PUFA (WMD = −0.24; 95% CI −0.86 to 0.39; *I*^2^ = 47.4%; P_het_ = 0.076)
			Five studies [197,200,201,203,208]	RCT; 380 subjects	4 to 18 months 25–85 years	↔ OC with N-3 PUFA (WMD = −0.63; 95% CI −1.84 to 0.57; *I*^2^ = 43.9%; P_het_ = 0.129)
			Four studies [201,202,205,206]	RCT; 169 subjects	6 weeks to 12 months 47–78 years	↓ CTX with N-3 PUFA (WMD = −0.37; 95% CI −0.73 to −0.01; *I*^2^ = 94.8%; P_het_ = 0.000)
			Three studies [197,203,205]	RCT; 224 subjects	6 weeks to 12 months 25–85 years	↔ NTX with N-3 PUFA (WMD = −1.74; 95% CI −3.97 to 0.48; *I*^2^ = 65.8%; P_het_ = 0.054)
Abdelhamid et al., 2019 [209]	Total PUFA	Two meta- analyses of BMD outcomes	Three studies [197,200,210]	RCT; 245 subjects	12 to 18 months 25–80 years	↔ LS BMD with total PUFA (SMD (random) = 0.15 g/cm^2^; 95% CI −0.21 to 0.51; *I*^2^ = 44%)
			Three studies [197,200,210]	RCT; 245 subjects	12 to 18 months 25–80 years	↔ FN BMD with total PUFA (SMD (random) = 0.35 g/cm^2^; 95% CI −0.26 to 0.96; *I*^2^ = 79%)
		Four meta- analyses of BTM outcomes	Three studies [197,200,211]	RCT; 195 subjects	1 to 2 years 67.8 years	↔ OC (MD (random) = 0.52 μg/L; 95% CI −1.99 to 0.95; *I*^2^ = 45%)
			Two studies [197,200]	RCT; 102 subjects	12 to 18 months 68 years	↔ serum BSAP (MD (random) = 0.52 μg/L; 95% CI −1.99 to 0.95; *I*^2^ = 45%)
			Three studies [197,200,210]	RCT; 246 subjects	12 to 18 months 25–80 years	↔ PTH (MD (random) = 4.70 pg/mL; 95% CI −0.43 to 9.83; *I*^2^ = 41%)
			Two studies [200,210]	RCT; 203 subjects	12 to 18 months 73.3 years	↔ DPD/Cr (MD (random) = 0.28 nmol/nmol; 95% CI −0.23 to 0.78; *I*^2^ = N/A)
	N-3 PUFA	Two meta- analyses of BMD outcomes	Four studies [199,201,202,212]	RCT; 463 subjects	1 to 2 years 45–78 years	↔ LS BMD by 2.6% with N-3 PUFA (MD (random) = 0.03 g/cm^2^, 95% CI −0.02 to 0.07; *I*^2^ = 72%)
			Four studies [199,201,202,212]	RCT; 463 subjects	1 to 2 years 45–78 years	↔ FN BMD by 4.1% with N-3 PUFA (MD (random) = 0.04 g/cm^2^; 95% CI 0.0 to 0.08; *I*^2^ = 71%)
		Three meta- analyses of BTM outcomes	Three studies [201,203,213]	RCT; 213 subjects	6 months 66 years	↔ OC (MD (random) = 2.03 μg/L; 95% CI −2.31 to 6.36; *I*^2^ = 55%)
			Two studies [201,202]	RCT; 116 subjects	6 months to 1 year 60.1 years	↔ CTX (MD (random) = −0.03 ng/mL; 95% CI −0.10 to 0.04; *I*^2^ = 0%)
			Three studies [201,202,213]	RCT; 313 subjects	6 months to 1 year 60.8 years	↔ PTH (MD (random) = −3.85 pg/mL; 95% CI −18.53 to 10.82; *I*^2^ = 54%)
Sadeghi et al., 2019 [214]	Fish consumption	Four meta- analyses of bone fracture outcomes	Six studies [215,216,217,218,219,220]	Four prospective and two case–controls; 164,908 subjects	1 to 24 years (10.2) 20–89 years	↓ hip fracture risk with fish consumption (pooled effect size = 0.88; 95% CI 0.79–0.98; *I*^2^ = 57.9; P_het_ = 0.02)
	N-3 PUFA		Five studies [90,217,218,221,222]	Prospective and case–control; 261,878 subjects	7 to 24 years (13.95 except case–control) 20–96 years	↓ hip fracture with dietary N-3 PUFA intake (pooled effect size = 0.89; 95% CI 0.80–0.99; *p* = 0.02; *I*^2^ = 17.3%; P_het_ = 0.29)
	ALA		Three studies [217,218,222]	Prospective; 260,106 subjects	7.8 to 24 years (16.2) 20–79 years	↔ hip fracture risk with dietary ALA intake (pooled effect size = 1.01; 95% CI 0.90 to 1.13; *p* = 0.92; *I*^2^ = 70.6%; P_het_ = 0.01)
	EPA + DHA		Four studies [216,217,218,222]	Prospective; 265,151 subjects	7.8 to 24 years (15.0) 20–79 years	↔ hip fracture risk with EPA + DHA intake (pooled effect size = 0.91; 95% CI 0.81 to 1.03; *p* = 0.12; *I*^2^ = 0.0%; P_het_ = 0.61)
Mozaffari et al., 2018 [223]	Total fat	Seven meta- analyses of bone fracture outcomes	Five studies [88,89,90,222,224]	Two prospective and three case–controls; 145,468 subjects	8.2 years (N/A in case–control) 34–80 years	↔ all fracture risk (including hip and total fracture) with total dietary fat (pooled effect size = 1.31; 95% CI 0.95 to 1.79; *p* = 0.09; *I*^2^ = 81.8%; P_het_ = 0.0001)
			Three studies [89,222,224]	One prospective and two case–controls; 139,280 subjects	7.8 years (N/A in case–control) 40–80 years	↔ hip fracture risk with total dietary fat (pooled effect size = 1.52; 95% CI 0.84 to 2.74; *p* = 0.16; *I*^2^ = 83.2%, P_het_ = 0.0001)
	SFA		Three studies [90,222,224]	One prospective and two case–controls; 138,474 subjects	7.8 years (N/A in case–control) 50–80 years	↔ all fracture risk (including hip and total fracture) with SFA (pooled effect size = 1.46; 95% CI 0.84 to 2.55; *p* = 0.18; *I*^2^ = 81.3%; P_het_ = 0.001)
			Two studies [222,224]	One prospective and one case–control; 138,140 subjects	7.8 years (N/A in case–control) 50–80 years	↑ hip fracture with SFA (pooled effect size = 1.79; 95% CI 1.05 to 3.03; *p* = 0.03; *I*^2^ = 77.3%, P_het_ = 0.01)
	MUFA+ olive oil		Four studies [90,222,224,225]	One prospective, two case–controls, and one RCT; 139,344 subjects	6.5 years (N/A in case–control) 50–80 years	↔ all fracture risk (including hip and total fracture) with MUFA + olive oil intake (pooled effect size = 1.22; 95% CI 0.73 to 2.04; *p* = 0.44; *I*^2^ = 81.3%; P_het_ = 0.0001)
	MUFA		Three studies [90,222,224]	One prospective and two case–controls; 138,474 subjects	7.8 years (N/A in case–control) 50–80 years	↔ all fracture risk (including hip and total fracture) with MUFA (pooled effect size = 1.47; 95% CI 0.74 to 2.92, *p* = 0.27; *I*^2^ = 86.1%; P_het_ = 0.0001)
			Two studies [222,224]	One prospective and one case–control; 138,140 subjects	7.8 years (N/A in case–control) 50–80 years	↔ hip fracture risk with MUFA (pooled effect size = 1.97; 95% CI 0.91 to 4.28; *p* = 0.08; *I*^2^ = 87.7%; P_het_ = 0.0001)
Shen et al., 2017 [226]	N-3 PUFA	Three meta- analyses of BTM outcomes	Six studies [197,200,203,204,206,213]	RCT; 368 subjects	6 to 18 months 65.4 years	↔ BALP with omega-3 fatty acids (SMD = 0.08; 95% CI −0.29 to 0.12; *p* = 0.429; *I*^2^ = 0.0%; P_het_ = 0.900)
			Six studies [197,200,201,203,208,213]	RCT; 288 subjects	4 to 18 months 68.6 years	↓ OC with omega-3 fatty acids from (WMD = −0.86 ng/mL; 95% CI −1.68 to −0.04; *I*^2^ = 36.6%; P_het_ = 0.850)
			Three studies [201,204,206]	RCT; 164 subjects	3 to 12 months 61 years	↔ CTX with omega-3 fatty acids among postmenopausal women (WMD = 0 ng/mL; 95% CI −0.04 to 0.04; *p* = 0.899; *I*^2^ = 0.0%; P_het_ = 0.785)

ALA, α-linolenic acid; BALP, bone alkaline phosphatase; BMD, bone mineral density; BSAP, bone-specific alkaline phosphatase; BTM, bone turnover marker; CI, confidence interval; Cr, creatinine; CTX, C-terminal telopeptide cross-link of type 1 collagen; DHA, docosahexaenoic acid; DPD, deoxypyridinoline; EPA, eicosapentaenoic acid; FN, femoral neck; het, heterogeneity; LS, lumbar spine; MD, mean difference; MUFA, monounsaturated fatty acid; N, number; N-3 PUFA, omega-3 polyunsaturated fatty acid; N/A, not available; NTX, N-telopeptide of type 1 collagen; OC, osteocalcin; PTH, parathyroid hormone; PUFA, polyunsaturated fatty acid; RCT, randomized controlled trial; SFA, saturated fatty acid; SMD, standardized mean difference; WMD, weighted mean difference; ↑, increase; ↓, decrease; ↔, no effect.

**Table 7:** The effects of fats on bone outcomes in individual human studies.

Nutrient Type	Ref	Study Type	N of Subjects Study Design	Follow-Up Period Age	BMD and/or Bone Fracture and/or BTM Outcomes
TF	Kato et al., 2000 [88]	Prospective: New York University Women’s Health Study	5854 postmenopausal women TF intake (g/day): Q1: <57.2; Q2: 57.2–64.1; Q3: 64.1–69.2; Q4: 69.2–75.0; Q5: ≥75.0	0–12.4 years (8.6) 34–65 years	↔ wrist fractures and hip fractures with TF in the age-adjusted model ↑ all fractures by 24% in Q5 of TF intake in the multivariate model (vs. Q1)
TF	Michaëlsson et al., 1995 [89]	Case–control	1140 subjects TF intake (g/day): Q1: <39; Q2: 39–48; Q3: 49–60; Q4: >60	N/A 40–75 years (cases, 67.6; control, 67.7)	↔ fracture risk in Q4 of TF intake in the multivariate model (vs. Q1)
TF, MUFA, PUFA, SFA, MUFA/PUFA, N-3 PUFA and N-6 PUFA	Martínez-Ramírez et al., 2007 [90]	Case–control	334 subjects TF intake (g/day): Q1: <87; Q2: 87–97; Q3: 98–112; Q4: ≥112 MUFA intake (g/day): Q1: <39; Q2: 39–46; Q3: 47–54; Q4: ≥54 PUFA intake (g/day): Q1: <11; Q2: 11–14; Q3: 15–17; Q4: ≥18 SFA (g/day): Q1: <23; Q2: 23–28; Q3: 29–33; Q4: ≥34 MUFA/PUFA ratio: Q1: <2.8; Q2: 2.8–3.3; Q3: 3.4–3.9; Q4: ≥4.0 N-3 PUFA intake (g/day): Q1: <11; Q2: 11–14; Q3: 15–17; Q4: ≥18 N-6 PUFA intake (g/day): Q1: <11; Q2: 11–14; Q3: 15–17; Q4: ≥18	N/A ≥65 years (cases, 73.2; control, 71.2)	↔ risk of low-energy fractures in Q4 of TF, MUFA, SFA, and omega-3 FA intake in the adjusted model (vs. Q1) ↑ risk of low-energy fractures in Q4 of PUFA (by 488%) and omega-6 FA intake (by 241%) in the adjusted model (vs. Q1) ↓ risk of low-energy fractures by 80% with the highest ratio of MUFA/PUFA in the adjusted model (vs. Q1)
TF, SFA, MUFA and PUFA	Benetou et al., 2011 [93]	Prospective: EPIC study	29,122 subjects	8 years 60–86 years (64.3)	↔ hip fracture with TF, SFA, PUFA, and MUFA after multivariate adjustment
Evening primrose oil (EPO)	Bassey et al., 2000 [197]	Parallel RCT: double blind	85 healthy pre- and postmenopausal women Intervention (/day): Efacal (E): 40 g of evening primrose oil, 440 mg of fish oil, and 1 g of Ca; Control: 1 g of Ca	12 months Premenopausal: 25–40 years; Postmenopausal: 50–65 years (Efacal, 58 ± 4.6; control, 55 ± 4.6)	↑ TB BMD within groups among premenopausal women ↓ TB BMD within groups among postmenopausal women ↔ TB BMD between groups among pre- and postmenopausal women ↑ serum Ca within groups among premenopausal women ↑ PTH within the E group among premenopausal women ↓ OC and BSAP within the E group among premenopausal women ↔ urinary hydroxyproline and NTX within groups among premenopausal women ↔ serum Ca, PTH within groups among postmenopausal women ↓ urinary hydroxyproline within the E group among postmenopausal women ↓ u-NTX, OC, BSAP within groups among postmenopausal women ↔ serum Ca, PTH, OC, BSAP, urinary hydroxyproline, and NTX between groups
ALA	Dodin et al., 2005 [199]	Parallel RCT: double blind	179 menopausal women Intervention (g/day): Flaxseed: 40 of flaxseed (9.1 ALA); Placebo: 40 of wheat germs	12 months 45–65 years (flaxseed, 54.0 ± 4.0; placebo, 55.4 ± 4.5)	↓ LS BMD within groups ↔ LS BMD between groups ↔ FN BMD
GLA + EPA	Kruger et al., 1998 [200]	Parallel RCT	60 women with osteoporosis or osteopenia Intervention (/day): Treatment: 6 g of evening primrose oil (EPO) and fish oil (FO) (60% LA + 8% GLA + 4% EPA + 3% DHA); Control: 6 g of coconut oil (placebo); All subjects: 600 mg Ca	18 months 79.5 ± 5.56 years	↔ LS BMD within the treatment group ↑ FN BMD by 1.3% within the treatment group ↓ LS BMD by 3.2% and FN BMD by 2.1% within the placebo group ↑ fracture risk in the placebo group (vs. treatment) ↔ serum Ca ↓ serum P in the treatment group (vs. placebo) ↑ urine Ca within groups ↔ urine P within groups ↓ urine P in the treatment group (vs. placebo) ↓ OC, u-DPD, and 1,25(OH)_2_D within both groups ↑ PICP, BSAP within both groups ↔ 25(OH)D within both groups
EPA + DHA	Tartibian et al., 2011 [201]	Parallel RCT	79 healthy sedentary postmenopausal women Intervention (/day): Supplement (S): 1000 mg by capsule (180 mg of EPA + 120 mg of DHA) Exercise + supplement (E+S) Exercise only (E) Control (C): placebo	6 months (24 weeks) 58–78 years (S, 63.1 ± 7.5; E+S, 59.7 ± 2.3; E, 61.4 ± 6.9; C, 58.9 ± 8.1)	↑ LS BMD, FN BMD within the E+S group and S group ↑ LS BMD, FN BMD in the E+S group (vs. E; vs. S; vs. C) and S group (vs. C) ↔ LS BMD, FN BMD within the C group ↑ estrogen, OC, 1,25(OH)_2_D, and calcitonin within the E+S group ↓ TNF-α, IL-6, PGE_2_, CTX, and PTH within the E+S group ↑ estrogen, OC, 1,25(OH)_2_D, and calcitonin in the E+S group (vs. E; vs. S; vs. C) ↓ TNF-α, IL-6, PGE_2_, CTX, and PTH in the E+S group (vs. E; vs. S; vs. C) ↑ calcitonin within the S group ↓ TNF-α, PGE_2_ within the S group ↑ estrogen, 1,25(OH)_2_D, and calcitonin in the S group (vs. C) ↓ TNF-α, PTH in the S group (vs. E; vs. C) ↓ PGE_2_ in the S group (vs. C) ↔ OC, CTX within the S group ↔ serum Ca and P within and between groups
EPA + DHA	Vanlint et al., 2012 [202]	Parallel RCT: Double blind	37 sedentary postmenopausal osteopenic women Intervention (/day): DHA: 400 mg of DHA (algal oil); Control: placebo (corn oil); All subjects: Ca and vitamin D_3_ supplement	1 year 59.2 years	↔ LS BMD, TH BMD, and FN BMD between groups ↓ CTX within groups ↔ CTX between groups
N-3 PUFA	Dong et al., 2014 [203]	Parallel RCT: double blind	116 postmenopausal women Intervention (/day): n-3 LC PUFA: 1.2 g of fish oil capsules (EPA + DHA); Control: placebo capsule (olive oil); All subjects: 315 mg Ca, 1000 IU vitamin D_3_	6 months 75 ± 7 years	↓ BSAP, OC within the N-3 LC PUFA group ↔ BSAP, OC between groups
EPA + DHA	Fonolla-Joya et al., 2016 [204]	Parallel RCT: double blind	103 healthy postmenopausal women Intervention (/day): Treatment: 0.5 L of low-lactose skim milk (40 mg/100 mL EPA + DHA, 0.54 g/100 mL oleic acid); Control: 0.5 L of semi-skim milk	12 months 50–70 years (59.7 ± 5.8)	↔ 25(OH)D, BALP, OPG ↓ i-PTH and RANKL within groups
N-3 PUFA	Griel et al., 2007 [205]	Crossover RCT	23 subjects Intervention (/day): Average American diet (AAD, control): 34% TF; 13% SFA; 13% MUFA; 9% PUFA (7.7% LA, 0.8% ALA) Linoleic acid diet (LA): 37% TF; 9% SFA; 12% MUFA; and 16% PUFA (12.6% LA, 3.6% ALA) A-Linolenic acid diet (ALA): 38% TF; 8% SFA; 12% MUFA; and 17% PUFA (10.5% LA, 6.5% ALA)	6 weeks 49.3 ± 1.6 years (men: 48.6 ± 1.6; women: 58.3 ± 2.7)	↓ NTX within ALA ↔ NTX in the ALA group (vs. the AAD group) ↔ BSAP between groups
EPA + DHA	Hutchins-Wiese et al., 2014 [206]	Parallel RCT: double blind	30 postmenopausal breast cancer survivors Intervention (/day): Fish oil (FO): 4 g of EPA + DHA capsules; Control: placebo capsules; All subjects: 1000 mg of Ca, 800 IU vitamin D_3_	3 months 48–84 years (62)	↔ 25(OH)D, PTH ↓ DPD, P1NP, and BSAP within the FO group ↓ serum CTX, P1NP, and DPD within the control group ↓ DPD in the FO group (vs. control)
PUFA	Lappe et al., 2013 [207]	Parallel RCT: double-blind pilot study	58 subjects Intervention (/day): geniVida bone blend (GBB): 30 mg of genistein + 800 IU vitamin D3 + 150 µg of vitamin K1 + 1 g of PUFA Placebo: placebo	6 months 45–55 years	↑ Ward BMD in the GBB group (vs. the placebo group) ↓ FN BMD in the placebo group (vs. the GBB group) ↔ LS BMD, troch BMD, intertrochanter BMD, TH BMD, and TB BMD between groups ↑ BSAP, NTX at the 3 and 6 mo. time points in the GBB group (vs. placebo group)
LA + GLA and EPA + DHA	Van Papendorp et al., 1995 [208]	Intervention	40 osteoporotic subjects Intervention (g/day): Evening primrose oil (EPO): 4 of EPO Fish oil (FO): 4 of fish oil EPO+fish oil (EF): 4 of EPO + fish oil Olive oil (OO): 4 of olive oil (control)	16 weeks 80 ± 4 years	↑ OC in the EF group (vs. EPO) ↑ PICP within the FO group ↓ ALP within the FO and EF groups ↑ urinary Ca/Cr ratio in the FO group
Virgin olive oil (VOO) and nuts	Bulló et al., 2009 [210]	RCT	238 elderly people at high risk for CVD Intervention: MedDiet+virgin olive oil (EOO): Mediterranean diet + VOO 15 L/3 months; MedDiet+nuts: MedDiet + 29 g/day of mixed nuts Control: low-fat control diet	12 months men: 55–80 years; women: 60–80 years (MedDiet+VOO, 67.8 ± 6.5; MedDiet+ nuts, 68.4 ± 6.0; control, 67.8 ± 6.1)	↔ BMD ↔ serum Ca, ALP, BSAP, OPG, DPD:Cr, and urinary Ca between groups ↑ PTH in MedDiet+nuts group (vs. MedDiet+VOO; vs. control)
Virgin olive oil	Fernández-Real et al., 2012 [211]	Parallel RCT	127 community-dwelling men with T2DM and risk factors for cardiovascular disease Intervention (/day): MedDiet+virgin olive oil (VOO): MedDiet + >50 mL VOO; MedDiet+nuts: MedDiet + 30 g of nuts; Control: low-fat control diet	2 years Med+VOO, 67.9 ± 6.9 years; Med+nuts, 67.6 ± 6.0 years; control, 68.4 ± 6.0 years	↑ OC, P1NP within the MedDiet+VOO group ↔ OC, P1NP within the MedDiet+nuts and control groups ↓ CTX within groups ↔ serum Ca within the MedDiet+VOO group ↓ serum Ca in the MedDiet+nuts and control groups ↔ UcOC
EPA + DHA	Chen et al., 2016 [212]	Parallel RCT: double blind	168 subjects with knee osteoarthritis Fat intake with supplement (g/day) High dose: 4.5 of fish oil (EPA + DHA); Low dose: 0.45 of fish oil (EPA + DHA)	2 years >40 years (low dose, 61.1 ± 9.6; high dose, 60.8 ± 10.4)	↔ LS BMD, FN BMD after adjusting for multivariables
N-3 PUFA	Sharif et al., 2010 [213]	Parallel RCT	18 osteoporotic postmenopausal women Intervention (/day): Treatment: 900 mg n-3 PUFA; Control: placebo	6 months Treatment: 60 ± 5.6 years; control: 63 ± 8.92 years	↔ OC, BSAP, serum Ca, vitamin D, and PTH ↓ urine PD within the treatment group
Dietary habits	Appleby et al., 2007 [215]	Prospective	34,696 adults Exposure: dietary habit (meat eaters, fish eaters, vegetarians, and vegans)	5.2 years 20–89 years (46.6)	↔ fracture risk among meat eaters, fish eaters, vegetarians and vegans
EPA + DHA	Virtanen et al., 2010 [216]	Prospective: Cardiovascular Health Study	5045 subjects (1305 for BMD data) Exposure: Tuna/other fish (servings): Q1: <1/month; Q2: 1–3/month; Q3: 1–2/week; Q4: ≥3/week Fried fish (servings) T1: <1/month; T2: 1–3/month; T3: ≥1/week EPA + DHA (mg/day) Q1: <145; Q2: 145–229; Q3: 230–411; Q4: 412–519; Q5: >519	11.1 years ≥65 years (72.8 ± 5.6)	↔ FN BMD, TH BMD in quartiles of tuna/other fish, fried fish, and EPA + DHA intake ↓ FN BMD, TH BMD with higher EPA + DHA intake among those with LA intake above median ↔ FN BMD, TH BMD between higher and lower EPA + DHA intake among those with LA intake below median ↔ hip fracture risk with consumption of tuna/other fish, fried fish, and EPA + DHA
ALA, EPA, DHA, EPA + DHA, AA and N-6:N-3 FA ratio	Farina et al., 2011 [217]	Prospective: Framingham Osteoporosis Study	904 older adults Total n-3 PUFA intake (g/day): not shown ALA (g/day): Q1: not shown, Q4: 0.84 AA intake (g/day): not shown EPA + DHA intake (g/day): not shown	17 years (men: 10.4, women: 12.7) ≥20 years (~75)	↓ hip fracture risk on ALA in both genders ↓ hip fracture risk by 54% in Q4 of ALA intake (vs. Q1) ↓ hip fracture risk by 80% in Q4 of AA intake (vs. Q1) ↔ hip fracture risk in Q4 of EPA, DHA, and EPA + DHA (vs. Q1)
Total PUFA, total n-3, PUFA, EPA + DHA, ALA, total n-6, PUFA and LA	Virtanen et al., 2012 [218]	Prospective: The Nurses’ Health Study (NHS) and Health Professionals Follow-up Study (HPFS)	122,354 adults without osteoporosis Total PUFA intake (men/women) (g/day): Q1: 9.4/7.9; Q2: 11.3/9.4; Q3: 12.7/10.5; Q4: 14.2/11.8; Q5: 16.8/13.9 Total n-3 PUFA intake (men/women) (g/day): Q1: 1.0/0.9; Q2: 1.2/1.1; Q3: 1.4/1.2; Q4: 1.6/1.4; Q5: 1.9/1.9 EPA + DHA intake (men/women) (g/day): Q1: 0.09/0.07; Q2: 0.18/0.12; Q3: 0.26/0.18; Q4: 0.36/0.24; Q5: 0.57/0.37 ALA intake (men/women) (g/day): Q1: 0.8/0.7; Q2: 0.9/0.8; Q3: 1.1/0.9; Q4: 1.2/1.0; Q5: 1.5/1.2 Total n-6 PUFA intake (men/women) (g/day): Q1: 8.2/6.9; Q2: 10.0/8.3; Q3: 11.3/9.3; Q4: 12.7/10.4; Q5: 15.2/12.4 LA intake (men/women) (g/day): Q1: 8.2/6.8; Q2: 10.0/8.1; Q3: 11.3/9.1; Q4: 12.7/10.2, Q5: 15.2/12.1	24 years 30–75 years	↔ hip fracture in Q4 of total PUFA intake and all types of PUFA subtypes in both genders (vs. Q1) ↓ hip fracture by 19% in Q4 of LA in women (vs. Q1)
Fish	Suzuki et al., 1997 [219]	Case–control: Mediterranean Osteoporosis Study (MEDOS)	747 elderly Japanese people Fish intake (/week): Low: ≤2 times; Moderate: 3–4 times; High: >4 times	1 year 65–89 years (cases: 78.6 ± 6.5, control: 78.3 ± 6.3)	↓ hip fracture risk by 42% in moderate fish intake (vs. low) ↔ hip fracture risk in high fish intake (vs. low)
Fish	Fan et al., 2013 [220]	Case–control	1162 cases and controls Freshwater fish intake (men/women) (g/day): Q1: 2.69/3.00; Q2: 10.90/10.49; Q3: 17.89/20.76; Q4: 39.10/55.81 Sea fish intake (men/women) (g/day): Q1: 0.54/0.12; Q2: 10.90/10.49; Q3: 17.86/20.76; Q4: 39.10/55.81 Mollusca and shellfish intake (men/women) (g/day): Q1: 0.27/0.08; Q2: 1.83/0.73; Q3: 4.15/2.88; Q4: 16.04/11.15 Total fish intake (men/women) (g/day): Q1: 9.75/7.88; Q2: 22.85/20.95; Q3: 35.25/36.33; Q4: 70.15/73.42	3 years 55–80 years (71)	↓ hip fracture in Q4 of sea fish (by 69%), Mollusca and shellfish (45%) and total fish (53%) in adjusted model (vs. Q1) ↔ hip fracture with freshwater fish intake in adjusted model
SFA, MUFA, PUFA, N-3, N-6 FA, LA, AA, ALA, EPA, DHA and DPA	Harris et al., 2015 [221]	Prospective	1438 subjects Exposure: fish oil (SFA, MUFA, PUFA: n-3, n-6 FA, LA, AA, ALA, EPA, DHA, and DPA) IQR of PUFA intake (men/women) (%): T1: 36.2–37.5/35.8–37.3; T2: 38.3–38.8/38.0–38.6; T3: 39.6–40.5/39.1–40.2 IQR of N-3 PUFA intake (men/women) (%): T1: 7.11–8.42/6.87–8.14; T2: 9.78–11.2/9.12–10.3; T3: 12.8–15.5/12.1–15.0 IQR of EPA intake (men/women) (%): T1: 1.27–1.71/1.20–1.63; T2: 2.23–2.96/2.04–2.52; T3: 3.97–5.46/3.40–5.24	7 years 66–96 years	↓ osteoporotic fracture risk by 40% in T3 of PUFA intake (vs. T1) ↓ osteoporotic fracture risk by 34% in T3 of N-3 PUFA intake (vs. T1) ↓ osteoporotic fracture risk by 45% in T3 of EPA intake (vs. T1) ↔ osteoporotic fracture risk with SFA, MUFA, N-6 PUFA, LA, AA, ALA, DHA, and DPA intake in men ↔ osteoporotic fracture risk with all types of oil intake in women
TF, SFA, MUFA and PUFA	Orchard et al., 2010 [222]	Cohort study: The Women’s Health Initiative Observational Study and Clinical Trials	136,848 postmenopausal women TF (% of E): Q1: 3.89–25.97; Q2: 25.98–32.24; Q3: 32.25–37.87; Q4: 37.88–51.35 SFA (% of E): Q1: 1.25–8.28; Q2: 8.29–10.52; Q3: 10.53–12.77; Q4: 12.78–36.70 MUFA (% of E): Q1: 1.03–9.63; Q2: 9.64–12.17; Q3: 12.18–14.51; Q4: 14.52–48.50 PUFA (% of E): Q1: 0.71–5.16; Q2: 5.17–6.42; Q3: 6.43–7.89; Q4: 7.90–31.84	7.8 years 50–79 years (63 ± 7)	↔ hip fracture and total fracture in Q4 of total fat or MUFA intake after multivariate adjustment (vs. Q1) ↑ hip fracture by 31% in Q4 of SFA intake after multivariate adjustment (vs. Q1) ↔ total fracture in Q4 of SFA intake after multivariate adjustment (vs. Q1) ↔ hip fracture in Q4 of PUFA intake after multivariate adjustment (vs. Q1) ↓ hip fracture by 5% in Q4 of PUFA intake after multivariate adjustment (vs. Q1) ↔ hip fracture and total fracture in Q4 of n-3 FA, ALA, and EPA intake after multivariate adjustment (vs. Q1) ↔ hip fracture in Q4 of n-6 FA intake after multivariate adjustment (vs. Q1) ↓ total fracture by 6% in Q4 of n-6 FA intake after multivariate adjustment (vs. Q1)
TF, animal fat, plant fat, SFA, MUFA, PUFA and MUFA/SFA	Zeng et al., 2015 [224]	Case–control	1292 elderly Chinese people TF (case–control) (% of E): Q1: 20.6/20.2; Q2: 25.3/25.3; Q3: 29.0/28.7; Q4: 35.3/34.3 Fat from an animal source (case–control) (% of E): Q1: 8.3/7.9; Q2: 11.4/11.5; Q3: 14.8/14.8; Q4: 22.4/20.3 Fat from a plant source (case–control) (% of E): Q1: 8.0/8.4; Q2: 11.6/11.4; Q3: 14.3/14.7; Q4: 18.9/18.9 SFA (case–control) (% of E): Q1: 4.8/4.7; Q2: 6.1/6.1; Q3: 7.1/7.2; Q4: 9.4/9.0 MUFA (case–control) (% of E): Q1: 7.2/6.8; Q2: 8.9/9.1; Q3: 10.7/10.6; Q4: 13.5/13.0 PUFA (case–control) (% of E): Q1: 4.4/4.5; Q2: 5.6/5.8; Q3: 7.0/6.9; Q4: 8.6/8.7 Ratio of MUFA to SFA (case–control) (%): Q1: 1.3/1.2; Q2: 1.4/1.4; Q3: 1.5/1.5; Q4: 1.7/1.7 MUFA from an animal source (case–control) (% of E): Q1: 2.7/2.6; Q2: 3.8/3.9; Q3: 5.1/5.1; Q4: 8.3/7.2 MUFA from a plant source (case–control) (% of E): Q1: 2.8/2.8; Q2: 4.2/4.1; Q3: 5.4/5.5; Q4: 8.1/7.5	N/A 55–80 years (Men: Cases, 70; Control, 69.5; Women: Cases, 71.2; Control, 71.1)	↑ hip fracture in Q4 of TF intake by 92%, fat intake from animal sources by 160%, SFA intake by 95%, MUFA intake by 122% and MUFA intake from animal sources by 155% in all covariate-adjusted models (vs. Q1) ↔ hip fracture in Q4 of fat from plant sources, PUFA intake, ratio of MUFA to SFA and MUFA intake from plant sources in all covariate-adjusted models (vs. Q1) ↑ hip fracture by 487% in Q4 of TF among men (vs. Q1) ↑ hip fracture in Q4 of fat from animal sources by 609% among men and by 82% among women (vs. Q1) ↑ hip fracture in Q4 of SFA intake by 610% and MUFA intake by 455% among men (vs. Q1) ↔ hip fracture for ratio of PUFA to SFA among men ↔ hip fracture in Q4 of fat from plant sources, PUFA intake, and ratio of MUFA to SFA among both genders (vs. Q1) ↔ hip fracture on TF and SFA intake among women ↓ hip fracture by 59% in Q4 of ratio of PUFA to SFA among women (vs. Q1)
EVOO	García-Gavilán et al., 2018 [225]	Parallel RCT	870 subjects at high cardiovascular risk Intervention (/day): MedDiet+Extra virgin olive oil (EVOO): MedDiet + 50 g of EVOO; MedDiet+Nuts: MedDiet+30 g of mixed nuts; Control: advice on a low-fat diet	5.2 years (follow-up: 8.9 years) 55–80 years	↔ osteoporotic fracture risk in the MedDiet+EVOO group and MedDiet+Nuts group (vs. control) ↓ risk of osteoporosis-related fractures by 51% in T3 of EVOO consumption (vs. T1)
LCO3-PUFA (ALA, EPA and DHA)	Lavado-García et al., 2018 [227]	Cross-sectional	1865 Spanish pre- and postmenopausal women Exposure: LCO3-PUFA (ALA, EPA, and DHA)	N/A 20–79 years (54 ± 10)	↑ FN BMD with ALA, EPA, and DHA in total women and pre and postmenopausal women ↑ LS BMD with EPA and DHA in total women and premenopausal women ↔ LS BMD with ALA, EPA and DHA in postmenopausal women ↑ FN BMD with ALA, EPA and DHA in total and premenopausal women among normal women ↔ LS BMD and FN BMD with ALA, EPA and DHA in postmenopausal women among normal women ↑ LS BMD with EPA and DHA in total and premenopausal women among normal women ↑ FN BMD and LS BMD with total LCO3-PUFA in normal and osteopenic women ↔ FN BMD with total LCO3-PUFA in osteoporotic women ↑ LS BMD with total LCO3-PUFA in normal women ↔ LS BMD with total LCO3-PUFA in osteopenic women

1,25(OH)_2_D, 1,25-dihydroxyvitamin D; 25(OH)D,25-hydroxyvitamin D; ALP, alkaline phosphatase; AA, arachidonic acid; ALA, α-linolenic acid; BALP, bone alkaline phosphatase; BMD, bone mineral density; BSAP, bone-specific alkaline phosphatase; BTM, bone turnover marker; Ca, calcium; Cr, creatinine; CTX, C-terminal telopeptide cross-link of type 1 collagen; CVD, cardiovascular disease; DHA, docosahexaenoic acid; DPA, docosapentaenoic acid; DPD, deoxypyridinoline; EPA, eicosapentaenoic acid; FN, femoral neck; GLA, gamma-linolenic acid; PTH, parathyroid hormone; IL-6, interleukin 6; i-PTH, intact parathyroid hormone; IQR, interquartile range; LA, linoleic acid; LCO3-PUFA, long-chain omega-3 polyunsaturated fatty acid; LS, lumbar spine; MedDiet, Mediterranean diet; MUFA, monounsaturated fatty acid; N, number; n-3 FA, omega-3 fatty acid; n3-LC, omega-3 long chain; N-3 PUFA, omega-3 polyunsaturated fatty acid; n-6 FA, omega-6 fatty acid; N/A, not available; NTX, N-telopeptide of type 1 collagen; OC, osteocalcin; OPG, osteoprotegerin; P, phosphorus; P1NP, type 1 procollagen-N-propeptide; PD, pyridinoline; PGE_2_, prostaglandin E_2_; PICP, procollagen; PTH, parathyroid hormone; PUFA, polyunsaturated fatty acid; RANKL, receptor activator nuclear factor-kB ligand; RCT, randomized controlled trial; SFA, saturated fatty acid; TB, total body; TF, total fat; TH, total hip; TNF-α, tumor necrosis factor alpha; troch, trochanter; T2DM, type 2 diabetes mellitus; UcOC, undercarboxylated osteocalcin; u-DPD, urinary deoxypyridinoline; u-NTX, urinary N-telopeptide of type 1 collagen; Ward, Ward’s triangle; ↑, increase; ↓, decrease; ↔, no effect.
